# Safety assessment and gastrointestinal retention of orally administered cerium oxide nanoparticles in rats

**DOI:** 10.1038/s41598-024-54659-9

**Published:** 2024-03-07

**Authors:** Hyoung-Yun Han, Bo-Kyung Kim, Jinhyung Rho, Se-Myo Park, Mi-Sun Choi, Soojin Kim, Min Beom Heo, Young-Su Yang, Jung-Hwa Oh, Tae Geol Lee, Seokjoo Yoon

**Affiliations:** 1 Department of Predictive Toxicology, Korea Institute of Toxicology, 141 Gajeong-ro, Yuseong-gu, Daejeon, 34114 Republic of Korea; 2 Department of Human and Environmental Toxicology, University of Science & Technology, Daejeon, 34113 Republic of Korea; 3 Jeonbuk Branch Institute, Korea Institute of Toxicology, 30 Baekhak1-gil, Jeongeup, Jeollabuk-do 56212 Republic of Korea; 4 Nanosafety Metrology Center, Korea Research Institute of Standards and Science (KRISS), 267 Gajeong-ro, Yuseong-gu, Daejeon, 34113 Republic of Korea

**Keywords:** Cerium dioxide, Nanoparticles, Subchronic toxicity, Oral toxicity, NOAEL, Biotechnology, Health care

## Abstract

Cerium oxide nanoparticles (CeO_2_ NPs, NM-212) are well-known for their catalytic properties and antioxidant potential, and have many applications in various industries, drug delivery, and cosmetic formulations. CeO_2_ NPs exhibit strong antimicrobial activity and can be used to efficiently remove pathogens from different environments. However, knowledge of the toxicological evaluation of CeO_2_ NPs is too limited to support their safe use. In this study, CeO_2_ NPs were orally administered to Sprague Dawley rats for 13 weeks at the doses of 0, 10, 100, and 1000 mg/kg bw/day, followed by a four week recovery period. The hematology values for the absolute and relative reticulocyte counts in male rats treated with 1000 mg/kg bw/day CeO_2_ NPs were lower than those in control rats. The clinical chemistry values for sodium and chloride in the treated male rat groups (100 and 1000 mg/kg/day) and total protein and calcium in the treated female rat groups (100 mg/kg/day) were higher than those in the control groups. However, these changes were not consistent in both sexes, and no abnormalities were found in the corresponding pathological findings. The results showed no adverse effects on any of the parameters assessed. CeO_2_ NPs accumulated in the jejunum, colon, and stomach wall of rats administered 1000 mg/kg CeO_2_ NPs for 90 days. However, these changes were not abnormal in the corresponding histopathological and immunohistochemical examinations. Therefore, 1000 mg/kg bw/day may be considered the “no observed adverse effect level” of CeO_2_ NPs (NM-212) in male and female SD rats under the present experimental conditions.

## Introduction

Cerium oxide nanoparticles (CeO_2_ NPs, NM-212) are widely used as automobile exhaust gas purification catalysts, in semiconductor insulation layer polishing, display glass polishing, cosmetics, and solid fuel cells. CeO_2_ NPs are used as abrasives to flatten the surfaces of mirrors, ophthalmic lenses, electronic wafers, and lenses, and as corrosion inhibitors^1–4^. Furthermore, CeO_2_ NPs are key components of ultraviolet light-blocking materials and play an important role in the oil refining process. CeO_2_ NPs are most commonly used as additives (catalysts) in diesel to enhance its combustion efficiency^5,6^. Many efforts have been made to develop safe therapeutics using CeO_2_ NPs. Recent data suggest that CeO_2_ NPs may be used as scavengers of reactive oxygen species to protect against cardiomyopathy, neuronal toxicity, and radiation damage^7–10^. However, the potentially hazardous health effects of nanosized CeO_2_ are emerging issues, and publications on the toxicity of nanoparticles are rapidly increasing. Safety issues concerning CeO_2_ NPs have been increasing and adverse effects of CeO_2_ NPs are continuously reported^11,12^.

Furthermore, the impact of oral and inhalation exposure of CeO_2_ NPs has not been fully determined and has attracted much research interest, including studies on and reports of its adverse effects on cells and organisms^13–16^. Moreover, a few in vivo tests have suggested that the bioavailability of CeO_2_ NPs is too low to exert intrinsic toxicity, especially when nanoparticles are orally administrated^17,18^. Toxic doses of orally administered nanoparticles were relatively higher than those of generally known toxic chemicals, which may result from the low bioavailability and low concentration of CeO_2_ NPs in target organs^19^. In one study, mice were orally administered 500, 1000, or 2000 mg/kg CeO_2_ NPs for 14 days, and serum biochemistry, hematology, and histopathological analyses were conducted^20^. Thus, it is critical to evaluate the safety of CeO_2_ NPs by performing adequate toxicity studies, given the insufficient toxicological information on CeO_2_ NPs to support their safe use. In this regard, the use of CeO_2_ NPs (NM-212) can provide reference data for future studies with different exposure amounts and dosage administration schedules. Therefore, the objective of the present study was to evaluate the toxicity of orally dosed CeO_2_ NPs in male and female Sprague Dawley (SD) rats to secure reference data that may be helpful for relevant future studies. The present study was performed in compliance with the Good Laboratory Practice (GLP) of the Organization for Economic Cooperation and Development (OECD, 1997) and the Ministry of Food and Drug Safety (MFDS, Korea, 2018).

## Materials and methods

### Preparation and characterization of CeO_2_ NPs (NM-212) suspension

**Figure 1:**
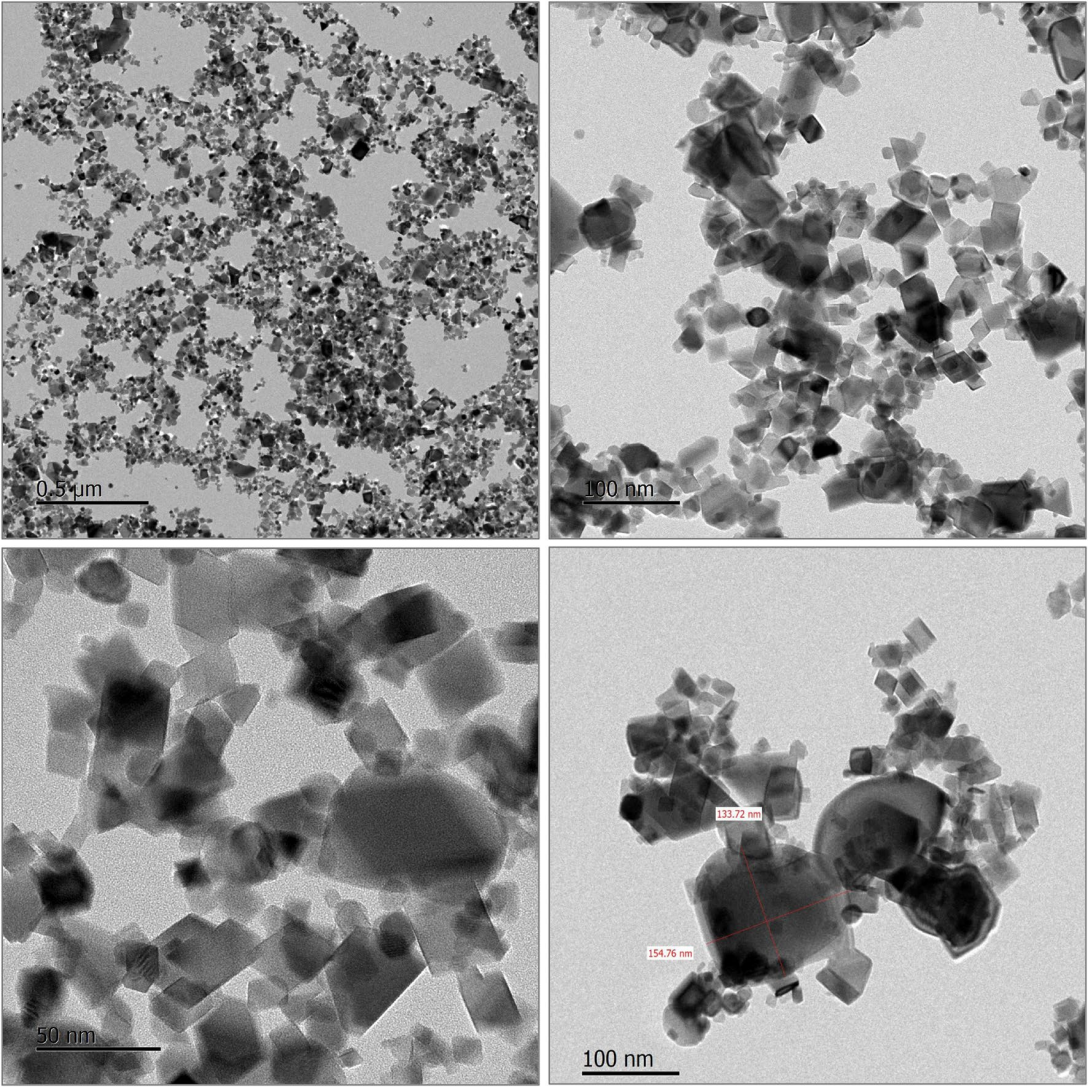
Characterization of CeO_2_ NPs.

**Figure 2:**
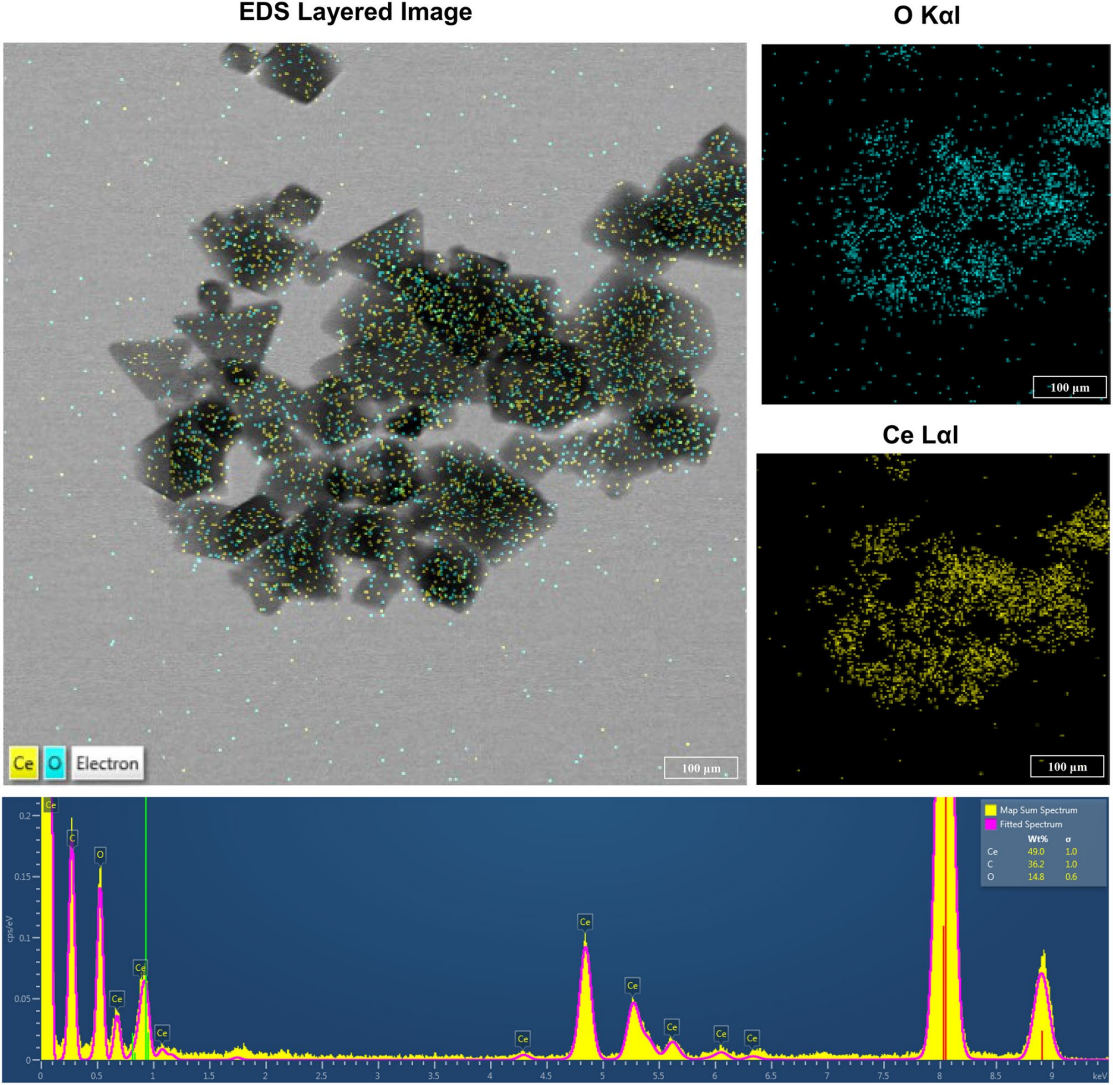
EDS analysis of CeO_2_ NPs.

CeO_2_ NPs (NM-212) were purchased from Sigma-Aldrich Chemie GmbH (Steinheim, Germany) (Product No. 22390; CAS No. 1306-38-3). CeO_2_ NPs were suspended in distilled water (DW) (stock concentration of 100 mg/mL) and the particle morphology was observed using TEM (JEM-3000F, 200 kV, JEOL Ltd., Tokyo, Japan) (Fig. 1). Energy-dispersive X-ray spectroscopy (EDS) analysis was also performed (JEM-2100F, JEOL Ltd., Tokyo, Japan) (Fig. 2). CeO_2_ NPs dispersion in DW is stable more than 5 days (Supplementary Information 3).

### Animal husbandry

Five-week-old male (n = 55) and female specific-pathogen-free outbred Sprague Dawley rats (n = 55) supplied by Orient Bio Inc. (Seongnam-si, Republic of Korea). The animals were acclimatized to their new surroundings for seven days. To reduce the body weigh variation blew 10% in each group, one hundred rats were selected and those animals randomly assigned to four groups (one control group and three treatment groups) using the Pristima system (Version 7.3 Xybion Medical System Co., USA). Ten males and 10 females/group were assigned to the four groups (main group), and 5 males and 5 females/group were assigned to the 0 mg/kg (control group) and 1000 mg/kg (high-dose). Those 2 groups (recovery group) were used to check the delayed toxicity or the recovered effects. All animals at terminal sacrifices were euthanized using isoflurane with necropsy. And, remained animals (5 males and 5 females) were euthanized using isoflurane. During the all-study periods, all animals were housed 2–3 per cage and there was attached cage card, described animal information, in each cage. The body weight ranges prior to the start of dosing were 204.6–234.5 g for the males and 158.1–194.1 g for the females. All rats were housed in polycarbonate cages with bedding (Laboratory animal Aspen bedding, Abedd Baltic Ltd., Jelgava, Latvia) throughout the study period and provided sterilized tap water and pelleted food (PMI Nutrition International, USA). The rats were maintained under the following conditions: 12/12 h light/dark cycle, temperature 23 ± 3 °C, relative humidity 50 ± 10%, air ventilation 10–20 times/h, and light intensity 150–300 lx. This study was performed in facilities approved by the Association for Assessment and Accreditation of Laboratory Animal Care International. The all experiments were reviewed and approved by the Association for the Assessment and Accreditation of Laboratory Animal Care (AAALAC) International and the Institutional Animal Care and Use Committee (IACUC) of the Korea Institute of Toxicology [approval no. 2006–0180] and all experiments were performed according to the ARRIVE guidelines, the guidelines published by the OECD and the GLP regulations for Nonclinical Laboratory Studies of Korea Food Drug Administration^21^. No linestatistical methods were used to predetermine sample sizes, but our sample sizes are based on to those reported in previous test study^22^. Animal experiments were designed to use all animals for the analysis, except for those that died or suffered from issues related to animal welfare throughout the experiment.

### Animal treatment

One hundred rats were divided into four groups, including a control group (n = 15, each sex), and three groups of orally administered CeO_2_ NPs (low dose: 10 mg/kg bw/day (n = 10, each sex), middle dose: 100 mg/kg bw/day (n = 10, each sex), high dose: 1000 mg/kg bw/day (n = 15, each sex). Dose formulations were administered by oral gavage once daily for 13 consecutive weeks. A daily oral dose of CeO_2_ NPs (NM-212) was administered to SD rats for 13 weeks (91 days), followed by a four week (28 days) recovery period. The animals of main group were sacrificed at Day 92 and animals of recorvery group were sacrificed at Day 119. Animals were dosed at a volume of 10 mL/kg. The dose volume was calculated based on the most recently measured body weight. Saline (vehicle control) was administered to the rats in the control group. Rats were divided into four groups and subjected to treatment doses of 0, 10, 100, and 1000 mg/kg bw/day CeO_2_ NPs, respectively. The effects of the CeO_2_ NPs were analyzed by clinical observation, body weight measurements, and recording of the rats’ food consumption. Serum chemistry and hematology examinations were performed to assess the clinical pathology of CeO_2_ NPs. In addition, during necropsy, gross observation, organ weight, histopathology, and immunohistochemistry were performed, and meaningful results were obtained in the stomach, jejunum, and colon.

### Mortality and body weight

All measurements and examinations were performed using the Pristima system. The general condition and behavior of the experimental animals were checked once daily throughout the acclimation period. However, during the treatment period and the day before necropsy was performed clinical signs were examined and recorded twice daily (before and after dosing). The animals were weighed on the day of arrival, prior to randomization, before the administration of the first dose, once weekly thereafter, and on the day of necropsy.

#### Food consumption and ophthalmic examination

Cage food consumption was recorded once during the acclimation period and once weekly during the treatment period. The individual food consumption was calculated and expressed as g/rat/day. External eye examinations were performed on all animals during the acclimation period. However, at week 13 before necropsy both external and fundus examinations of the animals in the control and high-dose (1000 mg/kg bw/day) groups were performed using a binocular indirect ophthalmoscope (Vantage Plus Digital, Keeler Ltd., England). Prior to examination via binocular indirect ophthalmoscopy, a mydriatic compound (Midrin-P; Santen Pharmaceutical Co., Ltd., Japan) was administered to each eye.

### Hematology analysis

All rats were fasted overnight before necropsy and blood samples were collected. Blood samples for hematological and clinical chemistry analyses were collected from the vena cava during necropsy. Samples for hematological analysis were collected in tubes containing EDTA-2 K. The following parameters were analyzed using an ADVIA 2120i hematology analyzer (Siemens, USA): white blood cell count, red blood cell count, hemoglobin concentration, hematocrit, mean corpuscular volume, mean corpuscular hemoglobin, mean corpuscular hemoglobin concentration, platelet count, differential leukocyte count (neutrophils, lymphocytes, monocytes, eosinophils, basophils, and large unstained cells), and reticulocyte count. In addition, blood samples treated with 3.2% sodium citrate were analyzed for prothrombin and activated partial thromboplastin times using an ACL Elite Pro coagulation analyzer (Instrumentation Laboratory, Italy).

### Biochemical analysis

Blood samples, simultaneously collected into tubes without anticoagulant for hematological and clinical chemistry analyses, were placed at room temperature for at least 90 min, and centrifuged at 3,000 rpm for 10 min at room temperature to obtain serum. The levels of glucose, blood urea nitrogen, creatinine, total cholesterol, total protein, albumin, albumin/globulin ratio, total cholesterol, triglycerides, phospholipids, aspartate aminotransferase, alanine aminotransferase, total bilirubin, alkaline phosphatase, gamma-glutamyl transferase, creatine kinase, calcium, inorganic phosphorus, sodium, potassium, and chloride were measured using an automatic analyzer (TBA 120FR NEO; Toshiba Corp., Tokyo, Japan).

### Gross findings and histopathological examination

After blood sampling, the animals were sacrificed by exsanguination through the vena cava and aorta under isoflurane anesthesia. Complete necropsy was performed on all experimental animals. The absolute weights of the brain, pituitary gland, liver, kidneys, spleen, heart, lungs, thymus, salivary gland, thyroid gland, testes, epididymis, prostate, seminal vesicle, uterus, and ovaries were determined, and relative organ weights (% of terminal body weight) were calculated.

Following detailed external and internal examinations, tissue samples (skin, mammary gland, testes, epididymis, prostate, seminal vesicle, urinary bladder, ovaries, uterus, vagina, spleen, pancreas, stomach, duodenum, jejunum, ileum, cecum, colon, rectum, kidneys, heart, lungs, adrenal gland, liver, salivary gland, mesenteric lymph node, mandibular lymph node, thyroid gland, aorta, thymus, trachea, tongue, esophagus, sciatic nerve, skeletal muscle, sternum/marrow, femur/joint/marrow, thoracic spinal cord, Harderian gland, brain, pituitary gland, and eyes/optic nerve) were collected from each animal and preserved in 10% neutral-buffered formalin. The eyes and optic nerves were fixed in Davidson’s fixative, whereas the testes were fixed in Bouin’s fixative for approximately 48 h and then transferred to 70% ethanol. Preserved tissues were obtained from animals in the control and high-dose groups.

### Immunohistochemistry and histopathological examination

Paraffin-embedded stomach and colon tissues were dewaxed using xylene and a graded alcohol series (100, 95, 70, and 50%). After washing with phosphate-buffered saline (PBS), the tissues were placed in an antigen retrieval solution (ENZO, Seoul, Korea) and permeabilized with PBS containing Tween-20 (PBST, 1%). After blocking with 5% (v/v) bovine serum albumin in PBST (0.01%), the tissues were incubated overnight at 4 °C with rabbit polyclonal antibodies against superoxide dismutase (SOD)-1, SOD-2 (Santa Cruz Biotechnology, Dallas, TX, USA), and cytochrome C (Cell Signaling Technology, Danvers, MA, USA). The tissues were then incubated with affinity-purified Alexa Fluor 488-conjugated goat anti-rabbit IgG (Invitrogen, Carlsbad, CA, USA) and mounted with 4′,6-diamidino-2-phenylindole mounting medium. Finally, images were captured using an inverted phase-contrast fluorescence microscope (IX51, Olympus, Tokyo, Japan). In addition, stomach and colon tissues were sectioned, stained with 0.5% periodic acid solution for 5 min, stained with Schiff's reagent for 15 min, and counterstained with hematoxylin solution for 2 min. All steps were performed at room temperature and the tissue sections were examined under a microscope.

### Statistical analysis

The data were statistically analyzed using multiple comparison methods. When the Bartlett’s test showed no significant deviations from variance homogeneity, analysis of variance (ANOVA) was used to identify differences among group means. A p value of p < 0.05 was considered statistically significant. The Dunnett’s test was used to determine differences between the control and treatment groups when the data were significant based on ANOVA. Furthermore, when significant deviations from variance homogeneity were observed based on the Bartlett’s test, a non-parametric comparison test, the Kruskal–Wallis (H) test, was performed to determine any group mean differences (p < 0.05). When a significant difference was observed in the Kruskal–Wallis (H) test, the Dunn’s rank sum test was performed to quantify the specific pairs of group data that significantly differed from the mean. The Fisher’s exact test was used to compare pairs of data (including prevalence and percentage). The probability level was set at 1% or 5%. Statistical analyses were performed by comparing the data for the different dose groups with those of the control group, using the Pristima system.

## Results

### Mortality, clinical observations, and body weight

No treatment-related mortality was observed and no treatment-related apparent adverse clinical findings were documented during the study period. The changes in body weight during the treatment period are shown in Table 1. No treatment-related changes in body weight were observed among the treatment groups (Supplementary Information 1).

**Table 1:** Absolute body weights in rats dosed with CeO_2_ NPs for 90 days (unit: g). Data are represented as mean ± SD.

Day	Male				Female			
	Control	10 mg/kg/day	100 mg/kg/day	1000 mg/kg/day	Control	10 mg/kg/day	100 mg/kg/day	1000 mg/kg/day
1	220.0 ± 7.23	221.5 ± 6.66	221.3 ± 5.84	223.0 ± 7.74	175.7 ± 11.00	175.5 ± 9.21	180.6 ± 8.74	177.2 ± 9.11
8	294.9 ± 9.73	292.1 ± 9.47	289.7 ± 11.91	288.6 ± 12.08	205.7 ± 13.78	204.6 ± 13.63	206.8 ± 12.69	202.4 ± 10.92
15	348.0 ± 21.40	340.4 ± 19.24	344.1 ± 21.08	339.0 ± 21.13	232.6 ± 16.55	231.1 ± 18.95	233.6 ± 17.60	226.4 ± 12.58
22	399.9 ± 23.00	397.6 ± 18.65	388.2 ± 27.34	389.2 ± 21.39	255.4 ± 19.92	248.2 ± 22.76	255.2 ± 16.91	247.4 ± 16.41
29	436.5 ± 29.75	436.5 ± 21.47	421.7 ± 30.51	421.1 ± 25.35	268.1 ± 25.58	264.3 ± 23.58	271.5 ± 21.13	264.8 ± 20.37
36	467.7 ± 31.88	468.7 ± 24.54	453.9 ± 36.32	448.2 ± 26.45	283.9 ± 23.94	275.0 ± 24.98	282.9 ± 23.70	274.2 ± 23.17
43	492.2 ± 36.34	497.8 ± 29.97	478.9 ± 41.90	477.1 ± 28.33	295.2 ± 24.51	290.7 ± 25.27	295.1 ± 26.12	283.6 ± 21.11
50	520.7 ± 41.31	522.0 ± 31.26	502.7 ± 44.75	496.5 ± 29.52	302.3 ± 26.82	294.5 ± 29.88	307.1 ± 29.69	294.2 ± 27.99
57	539.2 ± 44.40	539.4 ± 33.60	520.3 ± 49.10	513.2 ± 33.57	306.9 ± 31.32	300.8 ± 27.43	312.9 ± 24.80	304.4 ± 27.23
64	560.8 ± 47.59	562.3 ± 38.01	540.4 ± 51.20	530.6 ± 32.81	317.8 ± 30.38	307.5 ± 27.71	322.3 ± 29.35	307.8 ± 28.83
71	574.7 ± 51.22	576.4 ± 45.17	552.1 ± 53.18	545.9 ± 33.69	324.4 ± 29.41	318.0 ± 31.24	328.6 ± 32.12	312.8 ± 26.86
78	589.9 ± 52.39	594.6 ± 45.86	567.9 ± 54.03	561.0 ± 35.45	330.1 ± 32.74	321.9 ± 32.77	333.3 ± 29.92	320.8 ± 31.34
85	604.2 ± 54.23	607.3 ± 45.12	577.4 ± 54.36	573.4 ± 35.87	333.3 ± 33.92	324.3 ± 31.62	338.8 ± 30.50	328.0 ± 32.56
91	613.1 ± 54.04	617.5 ± 46.38	584.1 ± 57.15	580.0 ± 35.10	340.9 ± 30.61	331.6 ± 30.94	348.3 ± 31.37	329.2 ± 26.72

### Food consumption and ophthalmic examination

No changes or findings in food consumption (Table 2) were observed in any group (Supplementary Information 2). Abnormal ophthalmological findings were not observed in the treated group compared with the vehicle control group (data not shown).

**Table 2:** Changes in food consumption after treatment with CeO_2_ NPs during the test period (unit: g). Data are represented as mean ± SD.

Day	Male				Female			
	Control	10 mg/kg/day	100 mg/kg/day	1000 mg/kg/day	Control	10 mg/kg/day	100 mg/kg/day	1000 mg/kg/day
8	31.1 ± 1.22	31.6 ± 0.40	31.6 ± 2.43	32.0 ± 1.56	22.7 ± 0.69	22.2 ± 1.45	22.1 ± 0.69	22.3 ± 0.87
15	34.2 ± 1.53	33.4 ± 1.88	34.2 ± 2.68	34.2 ± 2.20	24.7 ± 1.13	23.6 ± 2.38	24.0 ± 0.71	23.7 ± 1.10
22	35.9 ± 1.11	35.7 ± 0.88	33.9 ± 2.23	37.0 ± 3.37	25.5 ± 1.36	23.9 ± 1.61	24.5 ± 0.58	25.2 ± 1.33
29	36.0 ± 1.48	35.9 ± 1.03	33.4 ± 2.82*	34.9 ± 3.07	25.4 ± 1.72	25.1 ± 1.12	25.1 ± 0.43	25.8 ± 1.72
36	35.2 ± 1.34	36.4 ± 1.56	34.3 ± 2.86	34.4 ± 2.43	25.7 ± 1.44	24.4 ± 1.22	25.2 ± 0.85	25.4 ± 1.01
43	35.8 ± 1.46	36.5 ± 1.52	34.4 ± 2.95	35.5 ± 1.81	25.5 ± 1.80	24.9 ± 1.69	25.4 ± 0.24	25.6 ± 1.51
50	35.8 ± 2.74	35.7 ± 1.65	34.0 ± 2.97	35.5 ± 1.90	25.0 ± 1.32	24.4 ± 1.64	25.3 ± 0.62	26.0 ± 1.55
57	35.3 ± 2.04	35.6 ± 1.76	33.8 ± 2.78	35.2 ± 1.74	24.5 ± 1.55	24.0 ± 1.88	24.2 ± 0.47	25.3 ± 1.44
64	35.2 ± 2.45	3.51 ± 1.56	33.6 ± 3.48	34.4 ± 1.72	24.2 ± 1.39	23.3 ± 0.94	24.4 ± 1.11	24.4 ± 0.94
71	38.1 ± 1.84	37.6 ± 1.98	35.8 ± 3.37	37.2 ± 1.95	25.3 ± 2.97	25.9 ± 2.49	27.1 ± 0.79	26.9 ± 2.08
78	38.4 ± 2.24	37.7 ± 1.81	36.0 ± 3.11	38.0 ± 2.33	26.9 ± 1.06	25.6 ± 2.53	26.7 ± 1.08	26.7 ± 1.63
85	36.9 ± 1.81	36.7 ± 1.34	34.5 ± 3.73	35.8 ± 1.66	25.6 ± 0.80	24.1 ± 2.14	25.2 ± 0.46	25.7 ± 1.56
91	35.4 ± 1.49	35.9 ± 1.02	33.3 ± 3.71	34.7 ± 1.80	25.0 ± 1.36	24.3 ± 2.82	26.1 ± 0.67	25.0 ± 0.87

* Significant differences from control group (p < 0.05).

### Hematology and serum biochemistry

Male rats treated with 1000 mg/kg bw/day CeO_2_ NPs exhibited a statistically significant decrease in the absolute and relative reticulocyte counts (%, 0.83-, and 0.83-fold, respectively).

A statistically significant increase in sodium levels (%, 1.01-, and 1.02-fold, respectively) was observed in male rats treated with 100 and 1000 mg/kg bw/day CeO_2_ NPs. Male rats treated with 1000 mg/kg bw/day CeO_2_ NPs exhibited a statistically significant increase in the level of chloride (%, 1.02-fold). Female rats treated with 100 mg/kg bw/day CeO_2_ NPs exhibited a statistically significant increase in the levels of total protein (%, 1.07-fold) and calcium (%, 1.05-fold) (Tables 3 and 4).

**Table 3:** Summary of hematological changes after dosing CeO_2_ NPs for 90 days. Data are represented as mean ± SD.

	Unit	Male				Female			
		Control	10 mg/kg/day	100 mg/kg/day	1000 mg/kg/day	Control	10 mg/kg/day	100 mg/kg/day	1000 mg/kg/day
RBC	(× 10^6/µL)	8.82 ± 0.405	8.80- ± 0.389	8.89 ± 0.475	8.83 ± 0.521	8.23 ± 0.223	8.07 ± 0.459	8.09 ± 0.363	8.23 ± 0.369
HGB	(g/dL)	15.2 ± 0.94	15.4 ± 0.75	15.6 ± 0.58	15.2 ± 0.49	15.1 ± 0.36	15.0 ± 0.50	15.1 ± 0.80	15.4 ± 0.60
HCT	(%)	50.0 ± 2.98	50.3 ± 2.54	51.1 ± 1.97	49.7 ± 1.74	48.4 ± 1.09	48.2 ± 2.11	48.8 ± 2.81	48.7 ± 1.88
MCV	(fL)	56.6 ± 1.82	57.1 ± 1.37	57.6 ± 2.64	56.4 ± 1.94	58.8 ± 0.96	59.8 ± 1.83	60.4 ± 1.80	59.2 ± 1.75
MCH	(pg)	17.3 ± 0.62	17.4 ± 0.48	17.6 ± 0.91	17.2 ± 0.69	18.3 ± 0.46	18.5 ± 0.63	18.7 ± 0.44	18.7 ± 0.47
MCHC	(g/dL)	30.5 ± 0.28	30.6 ± 0.50	30.5 ± 0.36	30.5 ± 0.50	31.2 ± 0.36	31.1 ± 0.45	31.0 ± 0.43	31.6 ± 0.48
RET	(%)	2.14 ± 0.211	2.03 ± 0.277	1.93 ± 0.254	1.79 ± 0.299*	1.96 ± 0.314	2.05 ± 0.284	1.97 ± 0.266	1.92 ± 0.284
RETA	(10^9/L)	189 ± 21.1	178 ± 26.5	171 ± 19.9	158 ± 25.6*	162 ± 28.9	165 ± 20.7	159 ± 19.5	158 ± 22.5
PLT	(10^3^/µL)	1169.2 ± 219.81	1148.9 ± 90.70	1009.6 ± 302.83	1163.0 ± 131.78	1081.8 ± 130.16	1090.6 ± 184.16	1059.2 ± 84.91	1131.6 ± 70.55
NEU	(%)	16.1 ± 5.17	14.6 ± 35.4	15.1 ± 4.64	14.7 ± 4.81	15.2 ± 6.05	11.7 ± 3.79	12.5 ± 5.25	12.5 ± 3.75
LYM	(%)	77.5 ± 5.72	79.5 ± 3.91	79.2 ± 4.31	79.7 ± 5.82	79.5 ± 5.44	82.4 ± 4.78	81.7 ± 5.65	81.7 ± 3.96
EOS	(%)	1.5 ± 0.41	1.5 ± 0.39	1.1 ± 0.31	1.2 ± 0.54	1.4 ± 0.40	1.6 ± 0.24	1.5 ± 0.45	1.5 ± 0.51
MON	(%)	3.8 ± 1.20	3.8 ± 1.04	3.6 ± 0.77	3.3 ± 1.22	2.9 ± 0.82	3.2 ± 1.30	2.8 ± 0.66	3.0 ± 1.27
BAS	(%)	0.5 ± 0.12	0.4 ± 0.10	0.4 ± 0.13	0.4 ± 0.12	0.5 ± 0.17	0.5 ± 0.18	0.6 ± 0.23	0.5 ± 0.19
LUC	(%)	0.7 ± 0.26	0.6 ± 0.12	0.6 ± 0.22	0.7 ± 0.21	0.6 ± 0.39	0.7 ± 0.20	0.9 ± 0.40	0.8 ± 0.36
WBC	(× 10^3^/µL)	12.34 ± 4.137	11.77 ± 2.656	9.75 ± 2.241	10.10 ± 1.910	7.43 ± 2.068	6.74 ± 1.511	7.25 ± 2.257	7.13 ± 1.437

* Significant differences from control group (p < 0.05).

**Table 4:** Results of clinical chemistry analysis after treating CeO_2_ NPs for 90 days. Data are represented as mean ± SD.

	Unit	Male				Female			
		Control	10 mg/kg/day	100 mg/kg/day	1000 mg/kg/day	Control	10 mg/kg/day	100 mg/kg/day	1000 mg/kg/day
GLU	(mg/dL)	108.4 ± 16.25	101.9 ± 13.76	108.3 ± 26.23	108.0 ± 20.98	118.4 ± 15.70	114.5 ± 20.98	124.3 ± 29.65	115.2 ± 27.54
BUN	(mg/dL)	16.8 ± 1.76	19.0 ± 2.62	17.0 ± 2.01	16.7 ± 0.79	21.3 ± 3.65	20.4 ± 3.17	20.3 ± 3.39	20.5 ± 0.056
CREA	(mg/dL)	0.40 ± 0.017	0.41 ± 0.28	0.41 ± 0.033	0.41 ± 0.036	0.53 ± 0.061	0.49 ± 0.046	0.47 ± 0.046	0.50 ± 0.056
TP	(g/dL)	6.43 ± 0.288	6.35 ± 0.295	6.55 ± 0.358	6.38 ± 0.326	7.03 ± 0.359	7.15 ± 0.272	7.55 ± 0.484*	7.18 ± 0.449
ALB	(g/dL)	4.17 ± 0.221	4.11 ± 0.239	4.34 ± 0.230	4.19 ± 0.127	5.07 ± 0.253	5.09 ± 0.253	5.47 ± 0.354	5.06 ± 0.546
A/G	(ratio)	1.87 ± 0.247	1.85 ± 0.182	1.97 ± 0.191	1.96 ± 0.368	2.61 ± 0.270	2.54 ± 0.525	2.64 ± 0.225	2.44 ± 0.525
AST	(IU/L)	103.3 ± 10.87	122.8 ± 15.31	110.7 ± 19.03	114.5 ± 21.95	112.2 ± 22.86	118.1 ± 19.22	108.2 ± 20.02	113.3 ± 12.42
ALT	(IU/L)	29.0 ± 6.04	29.0 ± 4.05	30.4 ± 4.58	26.8 ± 3.73	24.9 ± 4.55	30.7 ± 15.24	36.2 ± 13.53	30.0 ± 8.97
TBIL	(mg/dL)	0.099 ± 0.0200	0.093 ± 0.0077	0.108 ± 0.089	0.098 ± 0.0129	0.135 ± 0.0155	0.140 ± 0.0220	0.139 ± 0.0265	0.139 ± 0.0211
GGT	(IU/L)	0.32 ± 0.225	0.54 ± 0.186	0.67 ± 1.184	0.26 ± 0.243	0.68 ± 0.377	0.68 ± 0.294	0.50 ± 0.279	0.70 ± 0.387
ALP	(IU/L)	256.3 ± 48.25	288.7 ± 66.22	252.3 ± 30.64	251.0 ± 43.47	161.0 ± 32.27	164.2 ± 61.28	157.6 ± 54.31	162.1 ± 77.41
TCHO	(mg/dL)	70.9 ± 13.46	72.6 ± 15.20	80.7 ± 22.07	68.5 ± 7.34	72.9 ± 11.54	74.6 ± 15.31	79.7 ± 9.44	84.2 ± 18.23
TG	(mg/dL)	38.6 ± 12.99	39.2 ± 16.67	36.0 ± 16.51	33.6 ± 9.29	27.2 ± 18.04	25.3 ± 9.34	29.2 ± 10.72	21.2 ± 11.24
Ca	(mg/dL)	10.36 ± 0.322	10.37 ± 0.322	10.74 ± 0.602	10.48 ± 0.335	10.95 ± 0.454	10.99 ± 0.264	11.52 ± 0.315*	11.39 ± 0.743
IP	(mg/dL)	8.41 ± 0.923	8.61 ± 0.950	8.72 ± 0.748	8.31 ± 0.855	7.29 ± 0.590	7.30 ± 0.970	7.73 ± 1.028	7.70 ± 1.506
K	(mmol/L)	8.17 ± 1.717	8.09 ± 1.466	8.20 ± 1.072	7.99 ± 1.621	6.76 ± 0.950	7.07 ± 0.688	7.33 ± 0.457	7.40 ± 0.678
CK	(IU/L)	611.0 ± 128.60	753.6 ± 197.12	577.1 ± 103.23	681.3 ± 296.47	575.4 ± 168.58	590.4 ± 197.78	425.6 ± 149.18	525.8 ± 140.50
PL	(mg/dL)	99.0 ± 15.27	101.0 ± 17.65	108.2 ± 24.85	97.0 ± 6.82	135.8 ± 17.31	138.0 ± 21.03	146.5 ± 17.10	145.3 ± 29.60
Na	(mmol/L)	141.8 ± 1.32	142.8 ± 1.32	143.5 ± 1.58*	144.2 ± 1.69 +	144.3 ± 1.34	144.4 ± 1.26	145.3 ± 1.06	144.6 ± 3.31
Cl	(mmol/L)	101.8 ± 0.92	102.7 ± 1.70	102.6 ± 1.17	104.2 ± 1.48 +	104.4 ± 0.97	104.7 ± 1.34	105.3 ± 1.64	105.6 ± 2.88

* Significant differences from control group (p < 0.05).

+ Significant differences from control group (p < 0.01).

### Organ weights and gross findings

No treatment-related changes in absolute or relative organ weights were observed in any of the experimental male or female rats (Tables 5 and 6). In addition, no treatment-related gross findings were observed at necropsy in any of the treated male or female rats.

**Table 5:** Absolute organ weights after CeO_2_ NPs treatment at 10, 100 and 1000 mg/kg/day for 90 days. Data are represented as mean ± SD.

Unit (g)	Male				Female			
	Control	10 mg/kg/day	100 mg/kg/day	1000 mg/kg/day	Control	10 mg/kg/day	100 mg/kg/day	1000 mg/kg/day
Adrenal glands	0.068 ± 0.0086	0.067 ± 0.0090	0.061 ± 0.0102	0.066 ± 0.0099	0.084 ± 0.0145	0.079 ± 0.0126	0.085 ± 0.0155	0.080 ± 0.0183
Brain	2.172 ± 0.0763	2.230 ± 0.1016	2.188 ± 0.1323	2.211 ± 0.0950	2.037 ± 0.0849	2.082 ± 0.1197	2.107 ± 0.1315	2.033 ± 0.0183
Heart	1.712 ± 0.1212	1.803 ± 0.1573	1.669 ± 0.1876	1.658 ± 0.1237	1.097 ± 0.0903	1.087 ± 0.1568	1.091 ± 0.1032	2.033 ± 0.0840
Kidneys	4.053 ± 0.5126	4.131 ± 0.4541	3.975 ± 0.4439	4.070 ± 0.2951	2.203 ± 0.2132	2.197 ± 0.2559	2.398 ± 0.3362	2.330 ± 0.2374
Liver	16.860 ± 3.0411	16.352 ± 1.7828	15.398 ± 2.1129	15.682 ± 1.6165	8.745 ± 0.8305	8.450 ± 0.8574	9.360 ± 1.2754	8.804 ± 0.6821
Spleen	0.995 ± 0.0871	1.048 ± 0.1229	0.867 ± 0.1573	0.924 ± 0.1491	0.603 ± 0.1009	0.565 ± 0.0737	0.641 ± 0.0626	0.590 ± 0.0756
Thymus	0.439 ± 0.0850	0.431 ± 0.0908	0.350 ± 0.0488	0.411 ± 0.0930	0.295 ± 0.0598	0.322 ± 0.0701	0.353 ± 0.0383	0.344 ± 0.0317
Thyroid and parathyroid glands	0.028 ± 0.0036	0.032 ± 0.0065	0.029 ± 0.0052	0.029 ± 0.0066	0.022 ± 0.0044	0.026 ± 0.0050	0.026 ± 0.0070	0.023 ± 0.0039
Lung	1.873 ± 0.1433	2.039 ± 0.2642	1.812 ± 0.1711	1.815 ± 0.1357	1.380 ± 0.1020	1.402 ± 0.1667	1.409 ± 0.1557	1.394 ± 0.1856
Testes	3.523 ± 0.2563	3.670 ± 0.2802	3.642 ± 0.2084	3.591 ± 0.2354	–	–	–	–
Ovaries	–	–	–	–	0.098 ± 0.0144	0.104 ± 0.0215	0.102 ± 0.0309	0.110 ± 0.0193
Uterus/cervix	–	–	–	–	0.784 ± 0.1917	0.791 ± 0.2525	0.797 ± 0.2056	0.696 ± 0.1631

**Table 6:** Relative organ weights after CeO_2_ NPs treatment at 10, 100 and 1000 mg/kg/day for 90 days. Data are represented as mean ± SD.

Unit (%Body)	Male				Female			
	Control	10 mg/kg/day	100 mg/kg/day	1000 mg/kg/day	Control	10 mg/kg/day	100 mg/kg/day	1000 mg/kg/day
Adrenal glands	0.0117 ± 0.00160	0.0115 ± 0.00187	0.0110 ± 0.00171	0.0121 ± 0.00174	0.0269 ± 0.00436	0.0255 ± 0.00320	0.0261 ± 0.00406	0.0264 ± 0.00659
Brain	0.3729 ± 0.03729	0.3824 ± 0.03504	0.4007 ± 0.03353	0.4015 ± 0.02103	0.6526 ± 0.0569	0.6812 ± 0.05664	0.6549 ± 0.06734	0.6679 ± 0.04506
Heart	0.2929 ± 0.02405	0.3082 ± 0.02809	0.3041 ± 0.01882	0.3007 ± 0.01810	0.3496 ± 0.01025	0.3527 ± 0.02700	0.3377 ± 0.02761	0.3467 ± 0.02291
Kidneys	0.6898 ± 0.05300	0.7050 ± 0.06246	0.7258 ± 0.06819	0.7393 ± 0.06153	0.7038 ± 0.06160	0.7172 ± 0.08006	0.7393 ± 0.06358	0.7624 ± 0.04956
Liver	2.8513 ± 0.25879	2.7856 ± 0.18070	2.7986 ± 0.18963	2.8362 ± 0.13656	2.7878 ± 0.15186	2.7518 ± 0.17018	2.8912 ± 0.30323	2.8847 ± 0.14370
Spleen	0.1712 ± 0.02525	0.1787 ± 0.01831	0.1585 ± 0.02835	0.1668 ± 0.01923	0.1921 ± 0.02619	0.1840 ± 0.01888	0.1996 ± 0.02826	0.1929 ± 0.01696
Thymus	0.0749 ± 0.01415	0.0731 ± 0.01228	0.0640 ± 0.00814	0.0740 ± 0.01323	0.0937 ± 0.01539	0.1046 ± 0.01866	0.1100 ± 0.01783	0.1131 ± 0.01330
Thyroid and parathyroid glands	0.0047 ± 0.00065	0.0055 ± 0.00087	0.0053 ± 0.00102	0.0053 ± 0.00120	0.0070 ± 0.00127	0.0084 ± 0.00126	0.0079 ± 0.00155	0.0074 ± 0.00118
Lung	0.3204 ± 0.02631	0.3497 ± 0.05824	0.3315 ± 0.03233	0.3290 ± 0.01874	0.4402 ± 0.01580	0.4558 ± 0.02690	0.4362 ± 0.04385	0.4572 ± 0.05995
Testes	0.6037 ± 0.06395	0.6287 ± 0.06835	0.6679 ± 0.06823	0.6513 ± 0.02851	–	–	–	–
Ovaries	–	–	–	–	0.0311 ± 0.00393	0.0341 ± 0.00831	0.314 ± 0.00838	0.0360 ± 0.00665
Uterus/cervix	–	–	–	–	0.2505 ± 0.05985	0.2587 ± 0.09123	0.2499 ± 0.08202	0.2302 ± 0.06139

### Histopathological examination (CeO_2_ NPs accumulation in the stomach wall)

The results of histopathological examination are shown in Table 7. In males, zona fasciculata vacuolation in the adrenal glands, mononuclear cell infiltration in the heart, tubule basophilia, cysts, mononuclear cell infiltration in the kidneys, mononuclear cell infiltration in the liver, alveolar macrophage aggregation in the lung (with bronchi), acinar cell atrophy, mononuclear cell infiltration in the pancreas, and mononuclear cell infiltration in the prostate were observed in the control and 1000 mg/kg bw/day CeO_2_ NPs groups. In females, mononuclear cell infiltration in the heart and alveolar macrophage aggregation, mononuclear cell infiltration, and pigmented macrophages in the lungs (with bronchi) were observed in the control and 1000 mg/kg bw/day CeO_2_ NP groups. The abovementioned histopathological findings were randomly distributed between the control and treated groups, and were considered spontaneous or incidental^23^. Thus, no consistent treatment-related histopathological lesions were found in either sex.

**Table 7:** Summary of microscopic findings at control and 1000 mg/kg/day-dosed rat for 90 days.

		Males		Females	
		Control	1000 mg/kg	Control	1000 mg/kg
Adrenal glands	Vacuolation, zona fasciculata	2(1 >)	1(1 >)		
Heart	Cardiomyopathy	2(1 >)	0(1 >)		
	Infiltration, mononuclear cell	0(1 >)	1(1 >)	1(1 >)	0(1 >)
Kidneys	Basophilia, tubules	4(1 >)	1(1 >)		
	Cyst(s)	2(1 >)	1(1 >)		
	Dilation, pelvis	1(1 >)	0(1 >)		
	Infiltration, mononuclear cell	3(1 >)	2(1 >)		
	Nephropathy, focal	0(1 >)	1(1 >)		
	Mineralization			1(1 >)	0(1 >)
Liver	Infiltration, mononuclear cell	4(1 >)	4(1 >)	1(1 >)	1(1 >)
Lung with bronchi	Alveolar macrophage aggregation	1(1 >)	1(1 >)	1(1 >)	1(1 >)
	Infiltration, eosinophil, perivascular	1(1 >)	0(1 >)		
	Pleural fibosis	1(1 >)	0(1 >)		
	Infiltration, mononuclear cell			0(1 >)	2(1 >)
	Pigmented macrophages			0(1 >)	2(1 >)
Pancreas	Atrophy, acinar cell	2(1 >)	3(1 >)		
	Infiltration, mononuclear cell	1(1 >)	1(1 >)		
Prostate	Infiltration, mononuclear cell	2(1 >)	6(1 >)		
Skin	Congestion/hemorrhage	1(2 >)	0(2 >)		
	Erosion/ulcer	1(2 >)	0(2 >)		
	Granulomatous inflammation	1(3 >)	0(3 >)		
Skin, inguinal	Erosion	1(1 >)	0(1 >)		
Testes	Atrophy, tubular	1(1 >)	0(1 >)		

Data were expressed as number of animals showing histological abnormalities (number of findings in individual).

**Figure 3:**
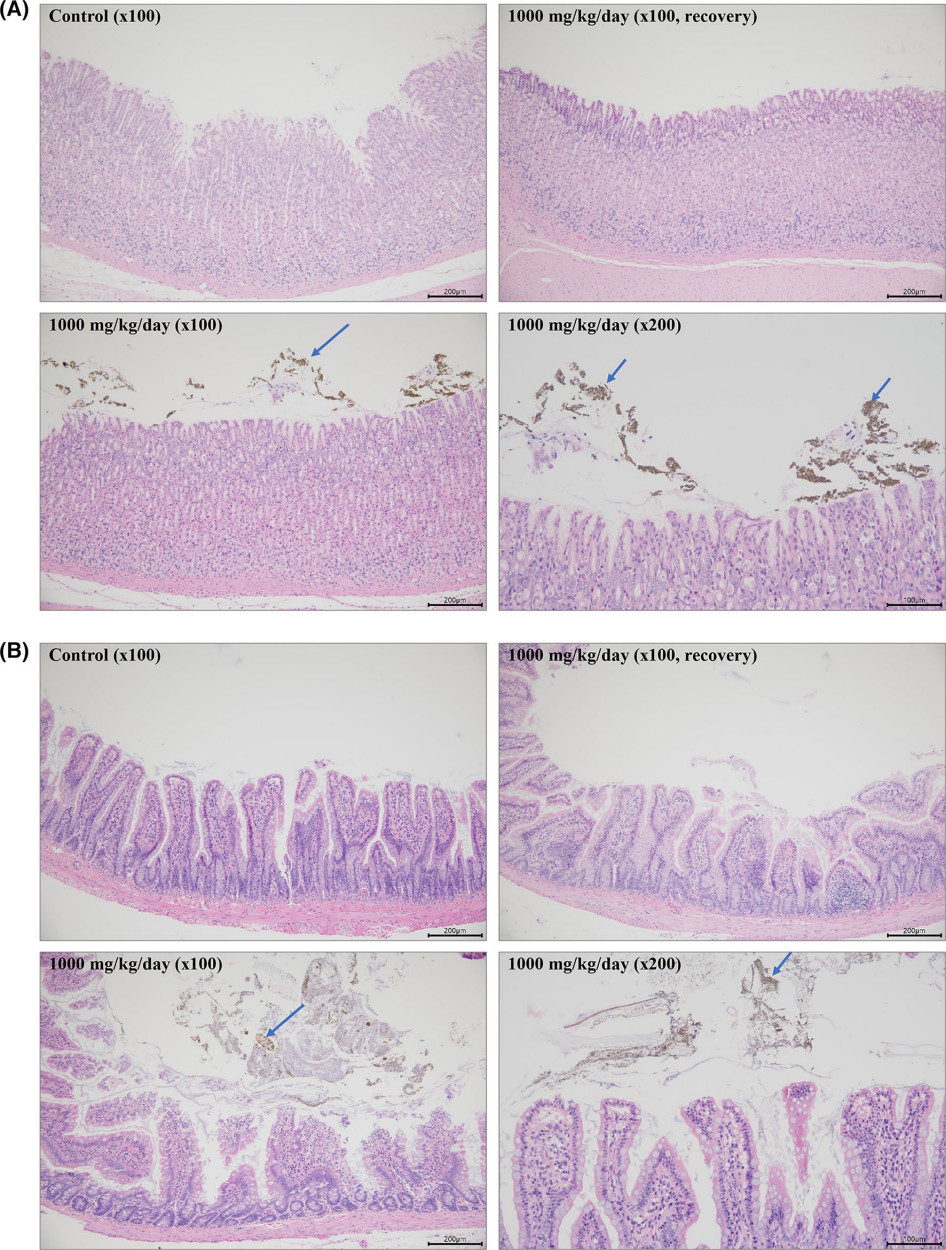

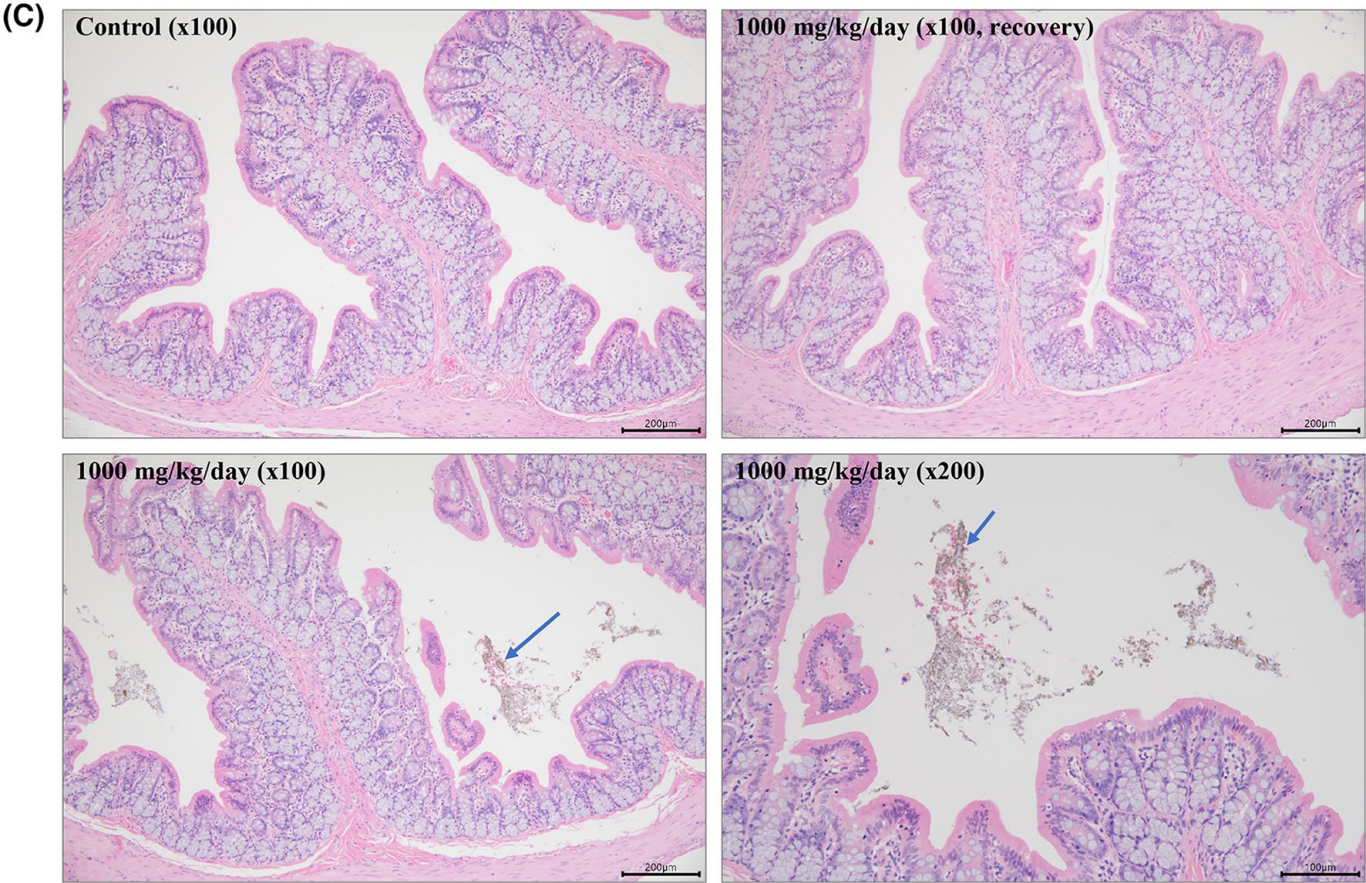
Histological findings in stomach (**A**), Jejunum (**B**), and colon (**C**). Paraffin sections were obtained from control and the highest dosing (1000 mg/kg/day) rats and stained with hematoxylin and eosin. No remarkable findings were obtained from the 0 mg/kg dosed rat (control) whereas CeO_2_ NPs accumulation was found at 1000 mg/kg dosed rat (Arrows: Accumulation of CeO_2_ NPs).

Accumulation of CeO_2_ NPs was observed in the jejunum, colon, and stomach wall of rats administered 1000 mg/kg CeO_2_ NPs for 90 days (Fig. 3).

### Immunohistochemical examination

**Figure 4:**
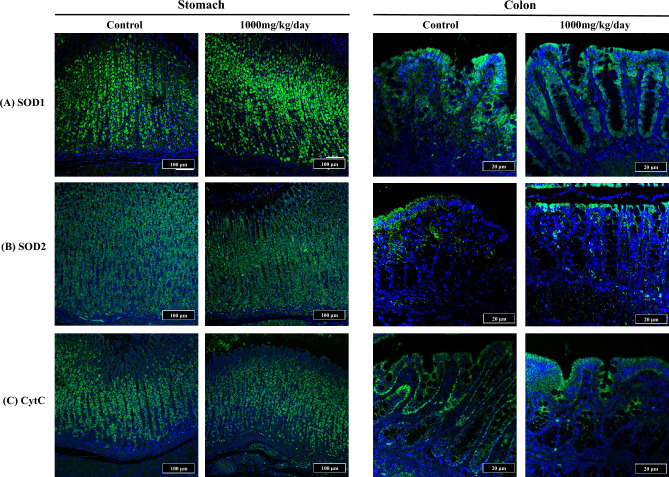
Periodic acid-Schiff stain (PAS) stained stomach (**A**) and colon (**B**) and a comparison of the number of goblet cell per crypt in colon between control and CeO2-administered rats (**C**). Arrows indicate accumulation of CeO2. Administering of CeO2 didn’t affect PAS-positive mucus layer in stomach and the number of goblet cell number.

In addition, as SOD plays a central role in inhibiting xenobiotic-induced oxidative damage and subsequent apoptosis, we assessed the effects of CeO_2_ NPs on the expression of SOD1, SOD2, and cytochrome C protein in the colonic tissues of rats in the control and 1000 mg/kg bw/day CeO_2_ NP groups. The expression of SOD1, 2, and cytochrome C are observed mainly in chief cells of stomach and apical border of epithelial cell in colon respectively. The expression levels of SOD1, SOD2, and cytochrome C proteins were not significantly different between the control and 1000 mg/kg bw/day CeO_2_ NP groups. (Fig. 4).

**Figure 5:**
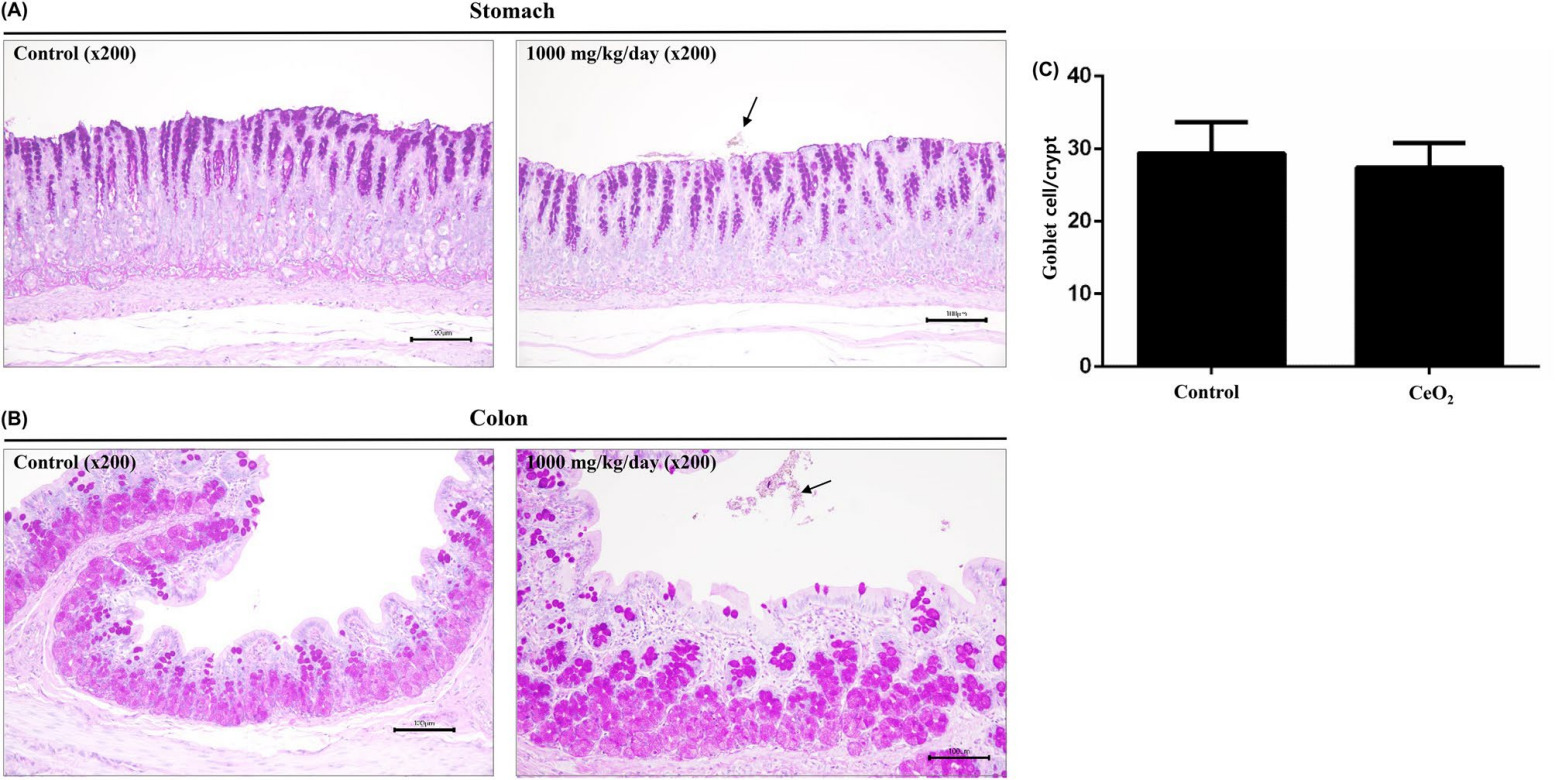
Immunofluorescence staining of stomach and colon tissue. Sections of control and highest dosing (1000 mg/kg) group were stained with (**A**) SOD1, (**B**) SOD2, and (**C**) Cytochrome C. The expression of SOD1, 2, and CytC are observed mainly in chief cells of stomach and apical border of epithelial cell in colon respectively. No specific difference were seen in expression of SOD1, SOD2 and CytC in stomach and colon between control and 1000 mg/kg-administered group respectively.

The number of goblet cells per crypt in the colon in the control and 1000 mg/kg bw/day CeO_2_ NPs groups were compared in periodic acid-Schiff stain (PAS)-stained stomach and colon tissues. PAS results showed that the addition of CeO_2_ NPs did not affect the PAS-positive mucus layer in the stomach or the number of goblet cells (Fig. 5).

## Discussion

CeO_2_ NPs have many applications in industry, drug delivery, and cosmetic formulations. They are mainly used for grinding glass, lenses, cathode ray tubes, jewelry, and complementary metal oxide semiconductor chips^24,25^. As CeO_2_ NPs in suspension have an excellent and well-controlled particle size distribution a very effective surface finish is achieved. Many efforts have been made to develop safe therapeutics using CeNPs^26–28^. Hence, the potentially hazardous health effects of nanoparticles are emerging issues among toxicologists and regulatory authorities. Safety issues concerning CeO_2_ NPs have been increasing and adverse effects of CeO_2_ NPs are continuously reported^11,12^.

In this study, we investigated the repeated toxicity of orally administered CeO_2_ NPs (NM-212) in rats to elucidate the in vivo toxicity mechanism of the nanoparticles. No dead animals were found during the experimental period (13 weeks after treatment). However, serum biochemical and hematological parameters showed statistically significant changes. The hematology values for the absolute and relative reticulocyte counts in male rats treated with 1000 mg/kg bw/day were lower than those in the control group. The clinical chemistry values for sodium and chloride in the treated male groups (100 and 1000 mg/kg/day) and total protein and calcium in the treated female groups (100 mg/kg/day) were higher than those in the control group. The changes in hematological and serum biochemistry values were of little toxicological significance, given the high variability in these values among rats, or among individual values in the same rat^29–31^. Other statistically significant changes in rats were not considered treatment-related because the degree of change was relatively small or the values were within the normal range. Moreover, histopathological analysis confirmed the relatively non-toxic level of CeO_2_ NPs used in this study^29–31^.

CeO_2_ NPs was reported to be toxicity by Kumari that the activity of serum ALP was found to be increased significantly at 300 and 600 mg/kg bw/day of CeO2 NPs after 28 days of repeated oral exposure in both male and female rats. And, significant damage of liver was observed as dilated portal tract with exposure to CeO_2_ NPs in 600 mg/kg dose groups. Splenic hyperplasia was found along with inflammation in brain tissue upon exposure with 600 mg/kg bw/day of CeO_2_ NPs^32^. The ALP is the foremost enzyme that is found to increase in liver disease. However, present study was not treatment-related changes in statistically significant for ALP enzyme. Additionally, the other parameters of serum biochemistry related to liver disease was not observed. And, our results was not observed with 1000 mg/kg bw/day of CeO_2_ NPs-related microscopic finding. In the histopathological study, positive findings, mild focal hemorrhage, and infiltration were randomly distributed between the control and treatment groups and were considered spontaneous or incidental^33–36^. Thus, no consistent treatment-related histopathological lesions were detected in either of the groups.

Meanwhile, CeO_2_ NPs accumulated in the jejunum, colon, and stomach wall of rats administered 1000 mg/kg bw/day CeO_2_ NPs for 90 days. Safety assessments after CeO_2_ NP administration have been conducted in previous studies. Most of the orally exposed CeO_2_ NPs were excreted through feces within 24 h. Other oral CeO_2_ NP administration studies suggested that oral CeO_2_ NPs could hardly be absorbed in the gastrointestinal tract, and that most of the orally administered CeO_2_ NPs were excreted in feces within a few days^13,37,38^. Our results also confirmed those of previous studies, showing that CeO_2_ NPs were not deposited in internal organs after repeated oral administration. Furthermore, we confirmed that CeO_2_ NPs did not accumulate in the internal organs of rats, and that most of the orally administered CeO_2_ NPs were excreted in the recovery animals. These changes were not observed in the corresponding histopathological and immunohistochemical analyses.

A significant lack of CeO_2_ NP toxicity was observed in our study, but these results do not imply that all sized-CeO_2_ NPs are safe for humans and the environment.

In conclusion, the results of our 13 week repeated-dose toxicity study in male and female SD rats revealed no treatment-related changes in mortality, clinical signs, body weight, food consumption, hematology, clinical chemistry, gross findings at necropsy, or organ weights. Furthermore, ophthalmic examination, urinalysis, histopathological, and immunohistochemical investigations showed no CeO_2_ NP-related changes. Therefore, 1000 mg/kg bw/day may be considered the ‘no-observed-adverse-effect-level’ of CeO_2_ NPs (NM-212) in male and female SD rats under the present experimental conditions. However, further investigations on the activities of CeO_2_ NPs, as well as the chronic toxicity of CeO_2_ NPs are warranted.

### Supplementary Information

Supplementary Information 1.

Supplementary Information 2.

Supplementary Information 3.

## Data Availability

The datasets used and/or analyzed during the current study are available from the corresponding author on reasonable request.
