# Supplementary material for: Safety assessment and gastrointestinal retention of orally administered cerium oxide nanoparticles in rats
Source: Sci Rep. 2024 Mar 7;14:5657. doi: 10.1038/s41598-024-54659-9 (PMC10920649; doi:10.1038/s41598-024-54659-9)
Supplement: Supplementary file 1 — Supplementary Information 1. [file 41598_2024_54659_MOESM1_ESM.pdf]

Appendix 3

Individual Body Weight

Individual Body Weight

Study: N220025

Rat/Sprague-Dawley REPEAT DOSE TOXICITY/TOX

Males Unit: g

Group #	Animal #	Treatment Day: 1 Session 1	Day: 8 Session 1	Day: 15 Session 1	Day: 22 Session 1	Day: 29 Session 1	Day: 36 Session 1	Day: 43 Session 1	Day: 50 Session 1
1 (V.C.)	1	231.0	312.5	345.5	427.7	483.9	518.9	549.9	583.7
	2	212.3	288.4	308.9	381.6	421.7	446.4	466.6	489.7
	3	219.8	282.8	307.7	369.5	397.3	437.3	463.6	491.4
	4	224.4	304.4	367.6	424.5	468.9	509.8	539.0	577.2
	5	224.9	303.1	372.3	435.2	479.7	489.8	530.9	568.0
	6	226.5	290.8	333.7	371.6	402.3	424.4	445.1	460.9
	7	231.3	296.5	361.3	408.1	447.9	486.3	511.3	538.3
	8	223.4	297.2	352.2	388.2	413.9	451.0	466.6	503.9
	9	224.8	300.1	378.2	428.8	472.7	510.1	535.0	558.2
	10	210.1	280.3	339.2	382.8	411.9	439.6	469.6	485.2
	11	211.1	279.1	328.6	377.8	415.4	446.1	464.8	485.4
	12	214.5	291.1	350.5	399.4	430.8	458.5	471.3	487.4
	13	219.0	291.0	347.2	377.0	405.0	426.8	443.3	480.0
	14	230.6	302.6	363.1	410.7	449.4	482.7	512.6	552.2
	15	226.6	303.1	364.7	416.0	447.1	488.5	512.8	549.6
2	16	224.6	294.9	339.9	389.5	421.3	443.4	472.9	486.4
	17	220.2	296.4	364.8	425.4	465.0	511.9	554.4	575.0
	18	209.1	272.8	331.6	375.4	413.2	445.6	471.7	501.6
	19	231.8	303.6	356.8	401.9	432.8	467.8	490.8	503.2
	20	215.3	279.3	335.7	376.7	406.0	443.2	462.7	488.9
	21	229.0	298.8	326.3	405.7	454.4	475.9	499.3	519.2
	22	221.1	297.4	311.0	384.8	428.8	450.2	479.9	506.3
	23	223.7	290.0	323.9	409.0	452.0	486.1	513.1	543.9

Individual Body Weight

Study: N220025

Rat/Sprague-Dawley REPEAT DOSE TOXICITY/TOX

Males Unit: g

Group #	Animal #	Treatment Day: 1 Session 1	Day: 8 Session 1	Day: 15 Session 1	Day: 22 Session 1	Day: 29 Session 1	Day: 36 Session 1	Day: 43 Session 1	Day: 50 Session 1
2	24	223.9	297.7	372.5	425.1	466.1	500.2	539.3	567.7
	25	216.7	290.2	341.8	382.5	425.4	462.4	493.7	527.5
3	26	215.7	289.6	347.5	396.2	430.4	459.7	484.2	507.4
	27	222.7	293.7	339.3	375.8	411.9	442.4	465.9	482.6
	28	219.9	286.4	327.5	371.6	401.2	423.2	440.5	455.8
	29	215.1	285.3	338.2	418.5	419.9	459.4	494.2	518.3
	30	224.9	299.6	366.1	383.1	439.0	486.9	516.2	556.9
	31	224.1	284.8	329.0	364.4	398.3	422.6	439.9	465.9
	32	225.1	285.3	337.2	377.9	411.3	440.6	456.3	476.0
	33	210.7	264.6	312.5	350.1	383.4	414.6	444.0	468.6
	34	229.5	298.6	359.1	400.9	427.3	451.9	472.9	498.2
	35	225.6	309.2	385.0	443.0	494.7	537.2	574.9	597.4
4	36	204.6	258.2	307.6	339.5	362.8	385.0	410.6	430.7
	37	223.2	278.2	327.1	372.3	400.7	424.7	451.2	469.9
	38	220.2	291.3	355.8	395.9	422.6	457.2	490.8	505.1
	39	212.2	283.5	356.0	410.7	455.0	473.5	503.0	516.0
	40	232.0	308.5	375.2	427.4	464.5	498.3	527.0	553.4
	41	222.5	295.3	317.1	400.9	433.3	454.3	486.7	502.7
	42	227.5	301.0	313.9	396.8	430.1	461.3	495.4	519.1
	43	220.0	287.0	306.5	380.9	418.7	450.7	479.7	500.3
	44	232.8	299.8	355.3	401.9	431.1	462.5	500.1	528.8
	45	224.4	299.9	365.6	410.9	440.0	460.0	481.2	494.4
	46	224.8	281.1	338.4	378.3	411.8	434.3	463.8	492.3

Individual Body Weight

Study: N220025

Rat/Sprague-Dawley REPEAT DOSE TOXICITY/TOX

Males Unit: g

Group #	Animal #	Treatment Day: 1 Session 1	Day: 8 Session 1	Day: 15 Session 1	Day: 22 Session 1	Day: 29 Session 1	Day: 36 Session 1	Day: 43 Session 1	Day: 50 Session 1
4	47	226.0	284.8	337.0	367.6	388.9	413.3	436.8	453.9
	48	234.5	292.5	344.5	379.9	408.7	447.2	475.6	495.2
	49	218.5	283.0	338.0	380.3	415.5	446.4	474.9	492.3
	50	221.4	285.6	347.3	395.4	433.3	454.8	479.1	493.8

Individual Body Weight

Study: N220025

Rat/Sprague-Dawley REPEAT DOSE TOXICITY/TOX

Males Unit: g

Group #	Animal #	Treatment Day: 57 Session 1	Day: 64 Session 1	Day: 71 Session 1	Day: 78 Session 1	Day: 85 Session 1	Day: 91 Session 1
1 (V.C.)	1	606.0	632.1	649.8	664.0	683.0	692.6
	2	500.9	520.4	537.4	543.2	557.9	577.4
	3	513.5	529.5	549.6	567.7	579.8	588.6
	4	599.6	619.2	643.8	660.5	674.6	689.4
	5	598.3	631.5	653.9	671.4	695.1	698.3
	6	476.2	501.4	499.3	511.1	523.4	533.1
	7	561.5	585.8	601.1	613.5	631.8	642.4
	8	520.4	545.2	546.2	566.3	580.0	587.0
	9	579.2	601.3	607.3	614.4	622.8	637.3
	10	503.9	527.7	537.3	565.3	570.8	582.1
	11	498.6	510.3	521.0	535.5	548.6	553.5
	12	498.7	521.7	539.9	545.0	563.0	569.8
	13	501.0	507.9	525.1	539.1	554.4	559.2
	14	572.3	592.1	601.1	623.1	639.0	643.3
	15	558.3	585.5	608.1	627.9	638.7	642.7
2	16	507.5	512.9	521.6	541.3	560.8	568.0
	17	600.9	629.6	666.0	684.4	700.0	708.9
	18	517.4	539.6	548.4	571.0	578.9	585.4
	19	530.8	550.3	560.7	575.3	596.4	607.1
	20	499.9	521.5	524.3	537.3	549.9	560.2
	21	533.6	569.4	587.4	623.4	627.4	645.5
	22	514.2	533.4	548.0	564.3	575.8	581.6
	23	566.5	590.1	604.2	615.0	623.7	635.4

Individual Body Weight

Study: N220025

Rat/Sprague-Dawley REPEAT DOSE TOXICITY/TOX

Males Unit: g

Group #	Animal #	Treatment Day: 57 Session 1	Day: 64 Session 1	Day: 71 Session 1	Day: 78 Session 1	Day: 85 Session 1	Day: 91 Session 1
2	24	582.3	607.0	619.1	635.0	648.0	658.6
	25	540.7	569.6	584.5	598.9	611.9	624.7
3	26	528.3	545.3	554.3	566.4	567.5	578.6
	27	499.7	516.1	528.4	545.7	556.3	563.7
	28	470.1	487.5	495.0	511.5	523.1	525.0
	29	542.0	571.8	583.8	604.0	612.0	630.5
	30	574.1	601.0	615.9	631.3	650.4	655.2
	31	484.9	499.6	511.7	525.3	537.1	541.4
	32	486.1	499.9	511.3	528.8	521.6	522.1
	33	478.8	502.4	515.8	530.2	544.0	546.8
	34	513.5	536.0	543.5	556.9	580.7	587.9
	35	625.8	644.1	661.0	679.2	680.9	689.8
4	36	439.9	462.6	482.7	492.9	498.1	505.8
	37	486.4	502.1	517.2	530.0	543.3	542.3
	38	526.4	543.8	565.2	575.5	583.1	584.1
	39	528.5	542.8	562.9	580.3	591.4	603.1
	40	583.8	606.1	621.5	634.3	645.3	647.7
	41	519.4	534.7	551.5	567.6	584.5	583.4
	42	539.2	551.3	570.6	585.0	595.4	597.0
	43	516.0	528.4	537.5	554.2	561.8	568.3
	44	543.3	565.0	579.2	603.7	612.8	626.0
	45	513.0	525.2	535.0	546.6	565.9	564.5
	46	510.4	528.7	545.8	561.3	581.8	595.3

Individual Body Weight

Study: N220025

Rat/Sprague-Dawley REPEAT DOSE TOXICITY/TOX

Males Unit: g

Group #	Animal #	Treatment Day: 57 Session 1	Day: 64 Session 1	Day: 71 Session 1	Day: 78 Session 1	Day: 85 Session 1	Day: 91 Session 1
4	47	462.1	487.9	495.1	503.4	514.9	534.3
	48	513.6	530.7	538.7	561.2	572.7	583.6
	49	508.4	530.1	550.1	562.1	575.5	578.0
	50	508.2	519.7	535.7	556.2	574.8	587.3

Individual Body Weight

Study: N220025

Rat/Sprague-Dawley REPEAT DOSE TOXICITY/TOX

Females Unit: g

Group #	Animal #	Treatment Day: 1 Session 1	Day: 8 Session 1	Day: 15 Session 1	Day: 22 Session 1	Day: 29 Session 1	Day: 36 Session 1	Day: 43 Session 1	Day: 50 Session 1
1 (V.C.)	51	166.4	187.3	217.7	235.5	239.3	258.9	271.6	276.3
	52	180.3	215.5	230.5	254.4	270.1	280.1	282.9	288.2
	53	167.4	200.4	232.2	253.8	256.8	278.2	291.5	299.5
	54	178.8	211.4	233.6	275.6	301.3	310.7	313.6	329.3
	55	187.4	215.9	251.3	270.8	264.0	293.0	306.7	313.7
	56	175.6	211.9	231.0	246.2	252.5	272.6	280.4	284.2
	57	163.8	188.6	211.8	226.4	233.4	252.3	262.3	264.3
	58	187.5	216.3	247.1	275.2	288.8	302.4	309.7	316.3
	59	174.1	207.1	243.4	250.7	288.2	289.1	314.2	315.5
	60	176.0	217.7	251.7	273.7	281.1	300.5	316.2	322.3
	61	181.4	210.0	239.2	280.4	307.3	319.4	332.8	355.0
	62	188.9	219.0	250.7	254.5	277.9	286.0	298.3	294.3
	63	158.1	180.2	198.4	216.5	221.3	231.0	245.2	251.0
	64	158.2	185.0	209.7	240.4	249.3	275.5	280.1	303.9
	65	192.0	219.7	240.7	277.1	289.5	309.3	322.6	320.0
2	66	174.1	207.4	240.9	262.2	275.2	300.4	319.2	319.3
	67	185.7	205.5	224.9	245.5	275.5	280.4	297.7	304.1
	68	168.3	186.1	209.5	213.8	237.1	245.9	260.9	254.6
	69	188.7	220.6	248.7	259.7	269.3	295.5	308.4	315.6
	70	160.9	188.8	204.0	219.7	222.7	238.1	252.9	247.1
	71	170.6	190.9	216.9	240.9	257.1	260.5	281.4	290.3
	72	179.0	210.1	247.0	259.1	279.7	290.7	300.1	306.5
	73	186.7	228.5	263.6	293.3	305.0	312.6	326.9	342.4

Individual Body Weight

Study: N220025

Rat/Sprague-Dawley REPEAT DOSE TOXICITY/TOX

Females Unit: g

Group #	Animal #	Treatment Day: 1 Session 1	Day: 8 Session 1	Day: 15 Session 1	Day: 22 Session 1	Day: 29 Session 1	Day: 36 Session 1	Day: 43 Session 1	Day: 50 Session 1
2	74	168.4	200.7	222.5	237.2	248.7	254.3	263.4	270.3
	75	172.9	207.8	232.8	250.1	273.1	271.3	295.9	294.9
3	76	186.3	208.5	234.4	251.2	265.4	260.7	282.3	287.0
	77	192.8	233.7	270.0	288.0	309.6	333.3	348.9	372.8
	78	163.3	189.8	214.3	239.3	242.4	261.1	270.4	286.0
	79	187.6	208.8	247.9	270.1	294.3	299.4	321.2	324.5
	80	178.8	206.4	222.0	248.2	263.8	275.2	276.0	294.9
	81	187.8	206.3	224.7	257.6	278.1	283.4	297.4	311.9
	82	180.2	213.2	238.5	262.6	281.1	294.6	294.5	308.9
	83	180.5	213.6	247.1	264.3	279.4	293.2	311.6	324.1
	84	177.3	196.0	220.8	232.5	246.5	252.7	262.4	263.5
	85	171.0	191.8	216.2	237.9	254.7	275.2	286.1	297.1
4	86	194.1	208.9	232.6	260.5	273.2	270.5	289.1	298.8
	87	177.8	204.9	229.5	263.0	283.2	298.4	303.7	318.3
	88	169.5	198.9	227.0	245.4	251.1	261.8	269.6	273.4
	89	193.1	221.9	238.6	272.3	291.4	305.7	304.0	323.7
	90	159.6	192.8	217.1	233.8	244.2	258.9	265.1	272.7
	91	176.8	194.7	224.3	242.6	256.9	255.2	279.1	289.5
	92	174.6	196.1	218.5	229.3	249.2	267.2	274.3	278.1
	93	180.7	207.3	227.5	250.6	274.2	290.4	288.2	303.4
	94	175.0	195.8	222.0	246.1	256.2	281.7	270.4	295.4
	95	170.5	187.4	210.0	225.3	237.5	235.5	253.4	263.0
	96	187.6	225.1	260.7	276.7	308.8	321.5	330.5	359.6

Individual Body Weight

Study: N220025

Rat/Sprague-Dawley REPEAT DOSE TOXICITY/TOX

Females Unit: g

Group #	Animal #	Treatment Day: 1 Session 1	Day: 8 Session 1	Day: 15 Session 1	Day: 22 Session 1	Day: 29 Session 1	Day: 36 Session 1	Day: 43 Session 1	Day: 50 Session 1
4	97	168.5	188.4	208.0	219.8	236.3	244.9	250.6	241.8
	98	176.6	207.3	232.3	254.5	275.0	285.0	300.3	309.6
	99	176.7	205.2	221.5	248.5	268.4	272.0	284.6	293.6
	100	176.2	201.4	226.0	242.5	265.8	264.5	291.8	292.8

Individual Body Weight

Study: N220025

Rat/Sprague-Dawley REPEAT DOSE TOXICITY/TOX

Females

Unit: g

Group #	Animal #	Treatment Day: 57 Session 1	Day: 64 Session 1	Day: 71 Session 1	Day: 78 Session 1	Day: 85 Session 1	Day: 91 Session 1
1 (V.C.)	51	282.5	299.6	321.9	303.7	308.4	324.5
	52	294.9	290.4	293.3	304.5	311.2	305.6
	53	297.4	313.3	318.9	320.5	326.2	342.2
	54	345.7	343.8	355.6	372.6	367.8	365.3
	55	308.8	327.9	338.9	339.0	330.4	348.1
	56	287.1	301.3	294.2	308.8	313.9	322.1
	57	266.4	276.4	283.3	289.2	291.4	301.4
	58	313.2	325.8	324.8	336.4	335.1	345.8
	59	336.5	349.3	343.4	365.5	367.7	357.2
	60	323.5	337.5	348.8	351.1	340.6	361.8
	61	363.2	374.6	380.4	395.1	407.1	405.6
	62	301.1	317.8	322.6	316.3	333.4	336.0
	63	241.2	255.3	270.4	272.8	267.2	282.7
	64	309.0	315.0	327.9	337.6	346.8	348.9
	65	332.4	339.5	341.6	338.0	352.7	366.5
2	66	322.1	341.3	351.7	349.1	343.2	347.5
	67	310.2	318.7	329.4	335.4	343.7	347.4
	68	268.8	275.0	272.1	268.2	285.2	286.7
	69	314.1	329.3	340.1	339.5	334.5	356.9
	70	257.4	258.7	268.4	275.6	270.3	285.4
	71	299.3	299.4	322.9	329.2	331.5	336.7
	72	315.9	315.3	332.0	341.6	346.7	357.0
	73	346.9	340.3	356.5	366.8	368.5	372.7

Individual Body Weight

Study: N220025

Rat/Sprague-Dawley REPEAT DOSE TOXICITY/TOX

Females Unit: g

Group #	Animal #	Treatment Day: 57 Session 1	Day: 64 Session 1	Day: 71 Session 1	Day: 78 Session 1	Day: 85 Session 1	Day: 91 Session 1
2	74	272.7	285.0	290.5	291.1	289.9	301.7
	75	300.4	312.0	316.5	322.6	329.4	323.5
3	76	288.6	295.2	304.5	315.0	314.4	322.8
	77	365.5	386.7	399.1	399.4	407.8	410.7
	78	297.3	308.2	323.8	320.0	338.2	344.2
	79	333.3	327.9	345.4	351.2	353.3	369.5
	80	306.4	306.6	300.5	316.1	320.8	317.0
	81	315.2	333.6	331.3	332.6	336.8	353.1
	82	311.5	319.6	321.5	337.2	340.2	344.1
	83	321.0	343.0	347.5	346.7	347.4	367.3
	84	275.5	279.5	281.5	284.8	289.8	299.1
	85	314.6	322.7	331.3	330.0	339.7	354.8
4	86	298.2	295.0	308.5	312.6	311.4	311.2
	87	329.8	336.2	331.0	343.5	354.5	347.6
	88	283.8	281.5	288.4	285.9	298.9	308.5
	89	325.7	331.7	335.2	341.5	351.2	351.1
	90	276.0	281.6	296.3	301.1	295.3	307.5
	91	301.8	295.5	299.4	312.3	318.4	325.6
	92	294.5	298.2	301.6	307.4	315.6	323.2
	93	315.4	330.5	328.0	348.2	357.9	358.9
	94	297.3	305.1	312.8	314.3	312.8	326.0
	95	270.3	268.2	276.4	282.4	286.1	290.7
	96	370.6	373.5	379.7	396.0	404.5	391.1

Individual Body Weight

Study: N220025

Rat/Sprague-Dawley REPEAT DOSE TOXICITY/TOX

Females Unit: g

Group #	Animal #	Treatment Day: 57 Session 1	Day: 64 Session 1	Day: 71 Session 1	Day: 78 Session 1	Day: 85 Session 1	Day: 91 Session 1
4	97	263.0	268.5	274.3	270.6	288.0	291.6
	98	325.9	330.1	335.9	337.8	354.8	345.9
	99	308.3	312.2	316.3	328.4	337.5	331.0
	100	305.6	309.5	308.9	330.6	332.8	327.4

Individual Body Weight

Study: N220025

Rat/Sprague-Dawley REPEAT DOSE TOXICITY/TOX

Males Unit: g

Group #	Animal #	Recovery Day: 1 Session 1	Day: 8 Session 1	Day: 15 Session 1	Day: 22 Session 1	Day: 28 Session 1
1 (V.C.)	11	546.1	550.0	570.3	586.5	598.3
	12	566.7	575.7	594.3	602.0	606.0
	13	558.1	555.9	572.5	571.3	581.4
	14	641.5	647.6	660.2	665.7	676.9
	15	640.3	656.3	669.3	679.5	679.8
4	46	587.7	592.8	610.5	623.4	629.7
	47	535.7	546.4	562.7	574.1	584.0
	48	575.6	589.9	614.7	625.3	625.3
	49	581.0	595.2	607.3	618.5	631.7
	50	586.0	601.5	615.0	633.0	646.4

Individual Body Weight

Study: N220025

Rat/Sprague-Dawley REPEAT DOSE TOXICITY/TOX

Females

Unit: g

Group #	Animal #	Recovery Day: 1 Session 1	Day: 8 Session 1	Day: 15 Session 1	Day: 22 Session 1	Day: 28 Session 1
1 (V.C.)	61	412.6	409.1	427.7	434.7	434.7
	62	338.6	345.0	346.2	352.2	362.3
	63	280.1	296.9	294.7	300.2	315.3
	64	339.0	346.6	360.2	359.6	367.2
	65	355.8	370.4	365.0	383.1	384.0
4	96	398.2	395.1	406.5	407.9	409.6
	97	292.4	294.8	287.6	306.5	308.2
	98	353.2	346.3	355.7	369.2	368.6
	99	337.6	337.1	341.7	347.1	343.8
	100	336.9	350.9	336.3	360.1	365.7
