# Supplementary material for: Safety assessment and gastrointestinal retention of orally administered cerium oxide nanoparticles in rats
Source: Sci Rep. 2024 Mar 7;14:5657. doi: 10.1038/s41598-024-54659-9 (PMC10920649; doi:10.1038/s41598-024-54659-9)
Supplement: Supplementary file 2 — Supplementary Information 2. [file 41598_2024_54659_MOESM2_ESM.pdf]

Appendix 4

Individual Food Consumption

Individual Food Consumption

Study: N220025

Rat/Sprague-Dawley REPEAT DOSE TOXICITY/TOX

Males

Unit: g/animal/day

Group #	Animal #	Pre-Treatment Day: 2	Treatment Day: 8	Day: 15	Day: 22	Day: 29	Day: 36	Day: 43	Day: 50
1 (V.C.)	1	22.8	30.7	32.1	36.9	34.8	34.7	35.9	34.4
	2	22.8	30.7	32.1	36.9	34.8	34.7	35.9	34.4
	3	22.8	30.7	32.1	36.9	34.8	34.7	35.9	34.4
	4	27.4	33.4	37.5	38.1	38.6	36.9	38.0	41.2
	5	27.4	33.4	37.5	38.1	38.6	36.9	38.0	41.2
	6	27.4	31.5	34.2	35.6	36.6	35.9	36.0	35.6
	7	27.4	31.5	34.2	35.6	36.6	35.9	36.0	35.6
	8	27.4	31.5	34.2	35.6	36.6	35.9	36.0	35.6
	9	23.6	29.2	34.2	35.2	35.4	34.3	35.1	34.3
	10	23.6	29.2	34.2	35.2	35.4	34.3	35.1	34.3
	11	24.9	30.3	33.9	35.2	34.2	33.3	33.4	32.8
	12	24.9	30.3	33.9	35.2	34.2	33.3	33.4	32.8
	13	24.9	30.3	33.9	35.2	34.2	33.3	33.4	32.8
	14	25.9	32.0	34.7	34.7	37.4	37.1	37.2	38.7
	15	25.9	32.0	34.7	34.7	37.4	37.1	37.2	38.7
2	16	26.5	31.6	34.4	36.0	36.8	38.1	37.6	37.0
	17	26.5	31.6	34.4	36.0	36.8	38.1	37.6	37.0
	18	26.5	31.6	34.4	36.0	36.8	38.1	37.6	37.0
	19	25.6	31.1	33.5	34.3	33.9	34.5	34.3	33.5
	20	25.6	31.1	33.5	34.3	33.9	34.5	34.3	33.5
	21	23.1	31.3	30.9	36.7	36.0	35.1	35.6	34.5
	22	23.1	31.3	30.9	36.7	36.0	35.1	35.6	34.5
	23	23.1	31.3	30.9	36.7	36.0	35.1	35.6	34.5

Individual Food Consumption

Study: N220025

Rat/Sprague-Dawley REPEAT DOSE TOXICITY/TOX

Males

Unit: g/animal/day

Group #	Animal #	Pre-Treatment Day: 2	Treatment Day: 8	Day: 15	Day: 22	Day: 29	Day: 36	Day: 43	Day: 50
2	24	26.6	32.3	35.9	35.3	36.3	37.6	38.3	37.7
	25	26.6	32.3	35.9	35.3	36.3	37.6	38.3	37.7
3	26	26.4	31.6	34.2	33.4	33.0	32.7	32.7	32.1
	27	26.4	31.6	34.2	33.4	33.0	32.7	32.7	32.1
	28	26.4	31.6	34.2	33.4	33.0	32.7	32.7	32.1
	29	25.7	30.7	33.4	33.3	31.0	34.6	35.5	35.5
	30	25.7	30.7	33.4	33.3	31.0	34.6	35.5	35.5
	31	24.3	29.2	31.5	31.9	31.8	32.0	31.8	31.4
	32	24.3	29.2	31.5	31.9	31.8	32.0	31.8	31.4
	33	24.3	29.2	31.5	31.9	31.8	32.0	31.8	31.4
	34	27.9	36.0	39.1	38.1	38.8	39.7	39.6	39.1
	35	27.9	36.0	39.1	38.1	38.8	39.7	39.6	39.1
4	36	23.4	29.5	31.5	32.4	30.0	30.4	32.7	33.0
	37	23.4	29.5	31.5	32.4	30.0	30.4	32.7	33.0
	38	23.4	29.5	31.5	32.4	30.0	30.4	32.7	33.0
	39	28.1	34.0	38.7	42.0	40.2	38.3	37.7	39.1
	40	28.1	34.0	38.7	42.0	40.2	38.3	37.7	39.1
	41	27.6	33.5	32.8	40.7	35.8	34.3	35.1	35.5
	42	27.6	33.5	32.8	40.7	35.8	34.3	35.1	35.5
	43	27.6	33.5	32.8	40.7	35.8	34.3	35.1	35.5
	44	26.0	32.9	35.4	37.3	36.4	36.7	38.1	36.6
	45	26.0	32.9	35.4	37.3	36.4	36.7	38.1	36.6
	46	23.1	31.7	34.2	35.1	33.8	34.1	35.7	36.0

Individual Food Consumption

Study: N220025

Rat/Sprague-Dawley REPEAT DOSE TOXICITY/TOX

Males

Unit: g/animal/day

Group #	Animal #	Pre-Treatment Day: 2	Treatment Day: 8	Day: 15	Day: 22	Day: 29	Day: 36	Day: 43	Day: 50
4	47	23.1	31.7	34.2	35.1	33.8	34.1	35.7	36.0
	48	23.1	31.7	34.2	35.1	33.8	34.1	35.7	36.0
	49	25.9	31.2	34.5	35.8	35.5	34.7	34.9	33.8
	50	25.9	31.2	34.5	35.8	35.5	34.7	34.9	33.8

Individual Food Consumption

Study: N220025

Rat/Sprague-Dawley REPEAT DOSE TOXICITY/TOX

Males

Unit: g/animal/day

Group #	Animal #	Treatment Day: 57	Day: 64	Day: 71	Day: 78	Day: 85	Day: 91
1 (V.C.)	1	35.2	35.1	38.3	38.1	36.7	35.8
	2	35.2	35.1	38.3	38.1	36.7	35.8
	3	35.2	35.1	38.3	38.1	36.7	35.8
	4	39.1	39.9	41.1	41.8	40.7	38.4
	5	39.1	39.9	41.1	41.8	40.7	38.4
	6	36.2	35.9	38.9	39.5	37.2	35.1
	7	36.2	35.9	38.9	39.5	37.2	35.1
	8	36.2	35.9	38.9	39.5	37.2	35.1
	9	34.4	33.6	36.5	36.7	35.8	34.8
	10	34.4	33.6	36.5	36.7	35.8	34.8
	11	32.2	31.8	35.3	35.1	34.6	33.3
	12	32.2	31.8	35.3	35.1	34.6	33.3
	13	32.2	31.8	35.3	35.1	34.6	33.3
	14	36.0	36.5	39.2	40.4	37.8	36.0
	15	36.0	36.5	39.2	40.4	37.8	36.0
2	16	37.3	36.7	38.9	39.4	37.3	35.7
	17	37.3	36.7	38.9	39.4	37.3	35.7
	18	37.3	36.7	38.9	39.4	37.3	35.7
	19	33.0	32.8	33.8	34.3	34.2	34.2
	20	33.0	32.8	33.8	34.3	34.2	34.2
	21	34.5	34.1	37.8	38.1	36.8	36.5
	22	34.5	34.1	37.8	38.1	36.8	36.5
	23	34.5	34.1	37.8	38.1	36.8	36.5

Individual Food Consumption

Study: N220025

Rat/Sprague-Dawley REPEAT DOSE TOXICITY/TOX

Males Unit: g/animal/day

Group #	Animal #	Treatment Day: 57	Day: 64	Day: 71	Day: 78	Day: 85	Day: 91
2	24	37.2	36.4	39.1	38.1	38.2	37.1
	25	37.2	36.4	39.1	38.1	38.2	37.1
3	26	32.8	31.6	33.7	34.4	32.1	31.4
	27	32.8	31.6	33.7	34.4	32.1	31.4
	28	32.8	31.6	33.7	34.4	32.1	31.4
	29	35.1	35.2	37.3	36.7	36.1	35.2
	30	35.1	35.2	37.3	36.7	36.1	35.2
	31	30.7	30.4	33.0	33.3	31.5	29.6
	32	30.7	30.4	33.0	33.3	31.5	29.6
	33	30.7	30.4	33.0	33.3	31.5	29.6
	34	38.4	39.6	41.7	41.7	41.1	39.5
	35	38.4	39.6	41.7	41.7	41.1	39.5
4	36	33.2	32.1	35.1	34.7	32.9	31.7
	37	33.2	32.1	35.1	34.7	32.9	31.7
	38	33.2	32.1	35.1	34.7	32.9	31.7
	39	38.2	37.2	41.0	42.4	37.9	36.4
	40	38.2	37.2	41.0	42.4	37.9	36.4
	41	34.6	34.1	36.0	38.3	36.4	34.0
	42	34.6	34.1	36.0	38.3	36.4	34.0
	43	34.6	34.1	36.0	38.3	36.4	34.0
	44	36.9	36.3	39.3	39.6	37.3	37.0
	45	36.9	36.3	39.3	39.6	37.3	37.0
	46	35.7	34.9	37.0	37.9	36.1	35.4

Individual Food Consumption

Study: N220025

Rat/Sprague-Dawley REPEAT DOSE TOXICITY/TOX

Males

Unit: g/animal/day

Group #	Animal #	Treatment Day: 57	Day: 64	Day: 71	Day: 78	Day: 85	Day: 91
4	47	35.7	34.9	37.0	37.9	36.1	35.4
	48	35.7	34.9	37.0	37.9	36.1	35.4
	49	33.3	32.8	36.5	36.6	35.0	35.1
	50	33.3	32.8	36.5	36.6	35.0	35.1

Individual Food Consumption

Study: N220025

Rat/Sprague-Dawley REPEAT DOSE TOXICITY/TOX

Females Unit: g/animal/day

Group #	Animal #	Pre-Treatment Day: 2	Treatment Day: 8	Day: 15	Day: 22	Day: 29	Day: 36	Day: 43	Day: 50
1 (V.C.)	51	18.5	22.8	24.9	25.7	24.8	24.4	24.2	24.7
	52	18.5	22.8	24.9	25.7	24.8	24.4	24.2	24.7
	53	18.5	22.8	24.9	25.7	24.8	24.4	24.2	24.7
	54	17.5	23.1	24.3	27.1	25.9	25.9	26.4	26.2
	55	17.5	23.1	24.3	27.1	25.9	25.9	26.4	26.2
	56	17.0	22.2	23.2	23.1	23.5	24.0	22.9	22.6
	57	17.0	22.2	23.2	23.1	23.5	24.0	22.9	22.6
	58	17.0	22.2	23.2	23.1	23.5	24.0	22.9	22.6
	59	18.9	24.0	27.0	26.9	28.9	28.3	28.4	26.3
	60	18.9	24.0	27.0	26.9	28.9	28.3	28.4	26.3
	61	18.4	22.6	25.1	25.9	26.5	25.9	26.4	25.6
	62	18.4	22.6	25.1	25.9	26.5	25.9	26.4	25.6
	63	18.4	22.6	25.1	25.9	26.5	25.9	26.4	25.6
	64	15.8	21.6	24.1	24.8	24.0	26.9	26.5	25.8
	65	15.8	21.6	24.1	24.8	24.0	26.9	26.5	25.8
2	66	18.6	20.0	21.5	22.2	25.1	24.6	24.5	23.8
	67	18.6	20.0	21.5	22.2	25.1	24.6	24.5	23.8
	68	18.6	20.0	21.5	22.2	25.1	24.6	24.5	23.8
	69	18.8	23.2	23.4	23.8	23.5	23.3	24.5	23.7
	70	18.8	23.2	23.4	23.8	23.5	23.3	24.5	23.7
	71	17.2	23.4	27.1	26.2	26.6	26.0	27.2	26.8
	72	17.2	23.4	27.1	26.2	26.6	26.0	27.2	26.8
	73	17.2	23.4	27.1	26.2	26.6	26.0	27.2	26.8

Individual Food Consumption

Study: N220025

Rat/Sprague-Dawley REPEAT DOSE TOXICITY/TOX

Females Unit: g/animal/day

Group #	Animal #	Pre-Treatment Day: 2	Treatment Day: 8	Day: 15	Day: 22	Day: 29	Day: 36	Day: 43	Day: 50
2	74	17.6	22.5	21.9	23.3	24.5	22.9	22.5	22.6
	75	17.6	22.5	21.9	23.3	24.5	22.9	22.5	22.6
3	76	16.7	23.0	24.7	24.8	25.6	24.1	25.3	25.5
	77	16.7	23.0	24.7	24.8	25.6	24.1	25.3	25.5
	78	16.7	23.0	24.7	24.8	25.6	24.1	25.3	25.5
	79	17.3	21.8	24.0	24.4	25.2	26.5	25.6	24.2
	80	17.3	21.8	24.0	24.4	25.2	26.5	25.6	24.2
	81	20.1	22.2	24.1	25.0	25.0	25.5	25.7	25.9
	82	20.1	22.2	24.1	25.0	25.0	25.5	25.7	25.9
	83	20.1	22.2	24.1	25.0	25.0	25.5	25.7	25.9
	84	20.5	21.0	22.7	23.4	24.4	25.2	25.1	25.0
	85	20.5	21.0	22.7	23.4	24.4	25.2	25.1	25.0
4	86	16.5	23.2	25.5	27.5	25.5	25.0	26.7	26.7
	87	16.5	23.2	25.5	27.5	25.5	25.0	26.7	26.7
	88	16.5	23.2	25.5	27.5	25.5	25.0	26.7	26.7
	89	18.0	22.9	23.9	25.5	26.3	25.0	24.3	25.6
	90	18.0	22.9	23.9	25.5	26.3	25.0	24.3	25.6
	91	15.3	21.9	22.5	23.7	23.8	24.2	24.8	24.5
	92	15.3	21.9	22.5	23.7	23.8	24.2	24.8	24.5
	93	15.3	21.9	22.5	23.7	23.8	24.2	24.8	24.5
	94	23.1	20.8	22.3	23.7	23.7	24.8	23.4	23.6
	95	23.1	20.8	22.3	23.7	23.7	24.8	23.4	23.6
	96	17.7	21.8	24.0	25.1	27.7	26.3	25.8	27.0

Individual Food Consumption

Study: N220025

Rat/Sprague-Dawley REPEAT DOSE TOXICITY/TOX

Females

Unit: g/animal/day

Group #	Animal #	Pre-Treatment Day: 2	Treatment Day: 8	Day: 15	Day: 22	Day: 29	Day: 36	Day: 43	Day: 50
4	97	17.7	21.8	24.0	25.1	27.7	26.3	25.8	27.0
	98	17.7	21.8	24.0	25.1	27.7	26.3	25.8	27.0
	99	22.7	23.4	23.7	25.1	28.2	27.2	28.3	28.6
	100	22.7	23.4	23.7	25.1	28.2	27.2	28.3	28.6

Individual Food Consumption

Study: N220025

Rat/Sprague-Dawley REPEAT DOSE TOXICITY/TOX

Females

Unit: g/animal/day

Group #	Animal #	Treatment Day: 57	Day: 64	Day: 71	Day: 78	Day: 85	Day: 91
1 (V.C.)	51	23.5	23.3	25.0	26.5	25.2	24.7
	52	23.5	23.3	25.0	26.5	25.2	24.7
	53	23.5	23.3	25.0	26.5	25.2	24.7
	54	26.3	24.6	29.4	28.1	25.5	26.0
	55	26.3	24.6	29.4	28.1	25.5	26.0
	56	22.3	22.1	22.5	25.0	24.3	22.6
	57	22.3	22.1	22.5	25.0	24.3	22.6
	58	22.3	22.1	22.5	25.0	24.3	22.6
	59	26.9	26.6	20.4	27.3	26.3	25.1
	60	26.9	26.6	20.4	27.3	26.3	25.1
	61	24.9	24.9	28.1	27.7	26.4	26.0
	62	24.9	24.9	28.1	27.7	26.4	26.0
	63	24.9	24.9	28.1	27.7	26.4	26.0
	64	24.1	24.6	26.7	27.4	26.5	26.6
	65	24.1	24.6	26.7	27.4	26.5	26.6
2	66	23.6	23.2	24.6	24.7	24.0	22.4
	67	23.6	23.2	24.6	24.7	24.0	22.4
	68	23.6	23.2	24.6	24.7	24.0	22.4
	69	23.3	23.0	24.2	25.1	23.3	25.8
	70	23.3	23.0	24.2	25.1	23.3	25.8
	71	26.5	24.5	29.6	29.1	26.9	27.7
	72	26.5	24.5	29.6	29.1	26.9	27.7
	73	26.5	24.5	29.6	29.1	26.9	27.7

Individual Food Consumption

Study: N220025

Rat/Sprague-Dawley REPEAT DOSE TOXICITY/TOX

Females Unit: g/animal/day

Group #	Animal #	Treatment Day: 57	Day: 64	Day: 71	Day: 78	Day: 85	Day: 91
2	74	21.4	21.8	23.8	22.2	20.9	20.5
	75	21.4	21.8	23.8	22.2	20.9	20.5
3	76	24.6	23.8	28.2	28.2	25.2	26.2
	77	24.6	23.8	28.2	28.2	25.2	26.2
	78	24.6	23.8	28.2	28.2	25.2	26.2
	79	23.8	22.7	26.5	26.6	24.3	24.9
	80	23.8	22.7	26.5	26.6	24.3	24.9
	81	23.7	25.5	26.4	25.5	25.6	26.7
	82	23.7	25.5	26.4	25.5	25.6	26.7
	83	23.7	25.5	26.4	25.5	25.6	26.7
	84	24.8	25.3	27.0	26.5	25.3	26.6
	85	24.8	25.3	27.0	26.5	25.3	26.6
4	86	25.5	24.2	28.4	26.8	25.7	25.3
	87	25.5	24.2	28.4	26.8	25.7	25.3
	88	25.5	24.2	28.4	26.8	25.7	25.3
	89	24.5	24.6	27.0	26.3	25.2	25.8
	90	24.5	24.6	27.0	26.3	25.2	25.8
	91	24.0	23.7	23.6	26.3	24.7	24.3
	92	24.0	23.7	23.6	26.3	24.7	24.3
	93	24.0	23.7	23.6	26.3	24.7	24.3
	94	23.3	22.7	24.8	23.8	23.5	24.0
	95	23.3	22.7	24.8	23.8	23.5	24.0
	96	26.4	25.2	28.1	27.0	26.4	24.3

Individual Food Consumption

Study: N220025

Rat/Sprague-Dawley REPEAT DOSE TOXICITY/TOX

Females Unit: g/animal/day

Group #	Animal #	Treatment Day: 57	Day: 64	Day: 71	Day: 78	Day: 85	Day: 91
4	97	26.4	25.2	28.1	27.0	26.4	24.3
	98	26.4	25.2	28.1	27.0	26.4	24.3
	99	27.8	25.7	29.3	30.0	29.0	26.6
	100	27.8	25.7	29.3	30.0	29.0	26.6

Individual Food Consumption

Study: N220025

Rat/Sprague-Dawley REPEAT DOSE TOXICITY/TOX

Males

Unit: g/animal/day

Group #	Animal #	Recovery Day: 8	Day: 15	Day: 22	Day: 28
1 (V.C.)	11	32.4	33.5	32.5	33.3
	12	32.4	33.5	32.5	33.3
	13	32.4	33.5	32.5	33.3
	14	36.7	36.6	35.2	34.2
	15	36.7	36.6	35.2	34.2
4	46	36.5	35.9	35.5	34.5
	47	36.5	35.9	35.5	34.5
	48	36.5	35.9	35.5	34.5
	49	35.4	36.0	36.0	35.7
	50	35.4	36.0	36.0	35.7

Individual Food Consumption

Study: N220025

Rat/Sprague-Dawley REPEAT DOSE TOXICITY/TOX

Females Unit: g/animal/day

Group #	Animal #	Recovery Day: 8	Day: 15	Day: 22	Day: 28
1 (V.C.)	61	25.9	26.1	25.7	27.4
	62	25.9	26.1	25.7	27.4
	63	25.9	26.1	25.7	27.4
	64	25.5	24.7	24.4	24.9
	65	25.5	24.7	24.4	24.9
4	96	24.7	24.6	24.8	25.5
	97	24.7	24.6	24.8	25.5
	98	24.7	24.6	24.8	25.5
	99	28.1	26.3	26.7	26.8
	100	28.1	26.3	26.7	26.8
