# Supplementary material for: Safety assessment and gastrointestinal retention of orally administered cerium oxide nanoparticles in rats
Source: Sci Rep. 2024 Mar 7;14:5657. doi: 10.1038/s41598-024-54659-9 (PMC10920649; doi:10.1038/s41598-024-54659-9)
Supplement: Supplementary file 3 — Supplementary Information 3. [file 41598_2024_54659_MOESM3_ESM.pdf]

CeO_2(NM-212) dispersion stability test (in DI-water)

- **Dispersion method**

: NIST special publication 1200-2, 1200-3

- **Dispersant :** DI water
- **Max. concentration :** 100 mg/ml
- **pH of NM-212 dispersion**
  - 100 mg/ml concentration : ~pH 3.5
  - 1 mg/ml concentration : ~ pH 4.5

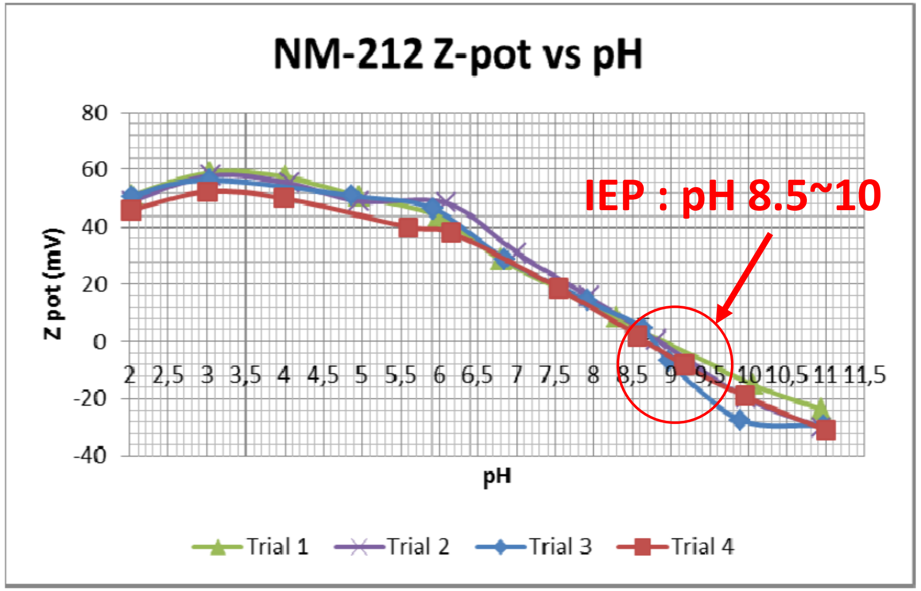

From NANoREG "protocol for the determination of zeta potential and IEP"

- **Size distribution measurement (by Malvern Zetasizer ZSP)**

  Before the measurement….
  - bath sonicated for 1 min
  - diluted to 1 mg/ml with DI-water

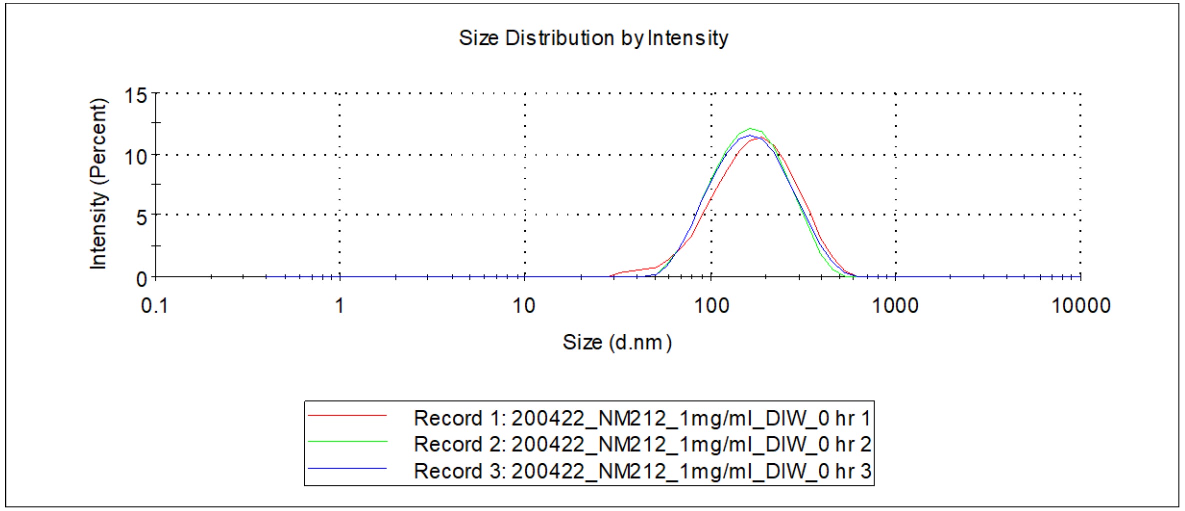

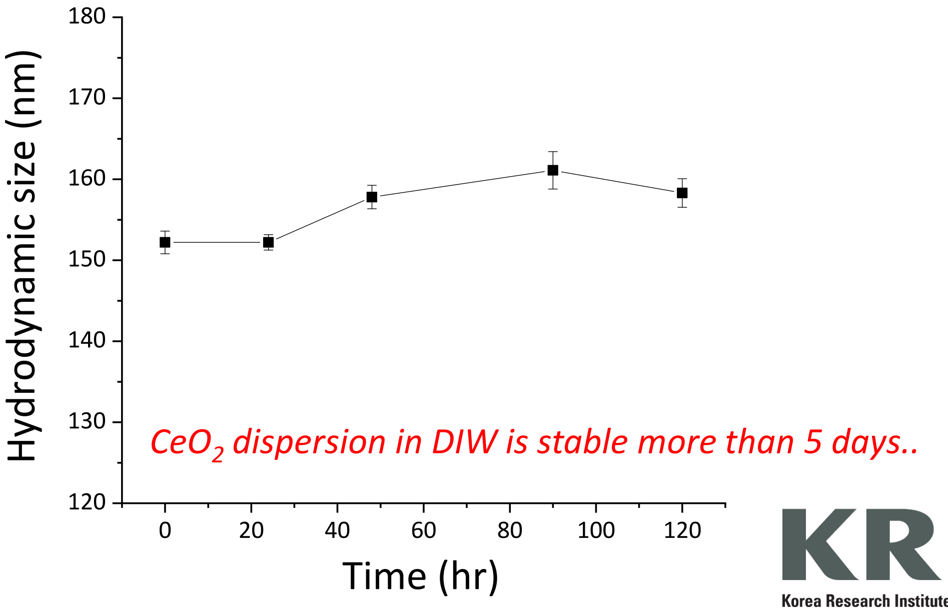

KRISS
Korea Research Institute of Standards and Science
